# Myosin V regulates synaptopodin clustering and localization in the dendrites of hippocampal neurons

**DOI:** 10.1242/jcs.230177

**Published:** 2019-08-22

**Authors:** Anja Konietzny, Judit González-Gallego, Julia Bär, Alberto Perez-Alvarez, Alexander Drakew, Jeroen A. A. Demmers, Dick H. W. Dekkers, John A. Hammer, Michael Frotscher, Thomas G. Oertner, Wolfgang Wagner, Matthias Kneussel, Marina Mikhaylova

**Affiliations:** 1 DFG Emmy Noether Group ‘Neuronal Protein Transport’, Center for Molecular Neurobiology, ZMNH, University Medical Center Hamburg-Eppendorf, 20251 Hamburg, Germany; 2 Institute for Synaptic Physiology, Center for Molecular Neurobiology, ZMNH, University Medical Center Hamburg-Eppendorf, 20251 Hamburg, Germany; 3 Institute of Structural Neurobiology, Center for Molecular Neurobiology Hamburg, ZMNH, University Medical Center Hamburg-Eppendorf, 20251 Hamburg, Germany; 4 Institute of Clinical Neuroanatomy, Faculty of Medicine, Theodor-Stern-Kai 7, 60590 Frankfurt, Germany; 5 Center for Proteomics, Erasmus MC, 3000 CA Rotterdam, The Netherlands; 6 Cell Biology and Physiology Center, National Heart, Lung Blood Institute, National Institutes of Health, Bethesda, MD 20814, USA; 7 Department of Molecular Neurogenetics, Center for Molecular Neurobiology, ZMNH, University Medical Center Hamburg-Eppendorf, 20251 Hamburg, Germany

**Keywords:** Synaptopodin, Spine apparatus, F-actin, Dendritic spines, Myosin

## Abstract

The spine apparatus (SA) is an endoplasmic reticulum-related organelle that is present in a subset of dendritic spines in cortical and pyramidal neurons, and plays an important role in Ca^2+^ homeostasis and dendritic spine plasticity. The protein synaptopodin is essential for the formation of the SA and is widely used as a maker for this organelle. However, it is still unclear which factors contribute to its localization at selected synapses, and how it triggers local SA formation. In this study, we characterized development, localization and mobility of synaptopodin clusters in hippocampal primary neurons, as well as the molecular dynamics within these clusters. Interestingly, synaptopodin at the shaft-associated clusters is less dynamic than at spinous clusters. We identify the actin-based motor proteins myosin V (herein referring to both the myosin Va and Vb forms) and VI as novel interaction partners of synaptopodin, and demonstrate that myosin V is important for the formation and/or maintenance of the SA. We found no evidence of active microtubule-based transport of synaptopodin. Instead, new clusters emerge inside spines, which we interpret as the SA being assembled on-site.

## Abstract (precis)

**Summary:** The spine apparatus is assembled locally in dendrites. This process, which relies on the presence of synaptopodin and F-actin, is disrupted by interfering with myosin V activity.

## INTRODUCTION

As an adaptation to their enormous size, neurons have developed a highly sophisticated trafficking system that mediates long-distance transport and local control of membrane and protein turnover (Hanus and Ehlers, 2016). Thus, most types of secretory membrane organelles, which are usually localized in the soma, can also be found in dendrites (Hanus et al., 2014; Mikhaylova et al., 2016). One key organelle is the endoplasmic reticulum (ER), which in all eukaryotic cells is important for lipid and membrane protein synthesis and transport, as well as for Ca^2+^ homeostasis. In neurons, the highly complex ER spans the entire cell, including soma, axon and dendritic tree, and is even found in dendritic spines (Toresson and Grant, 2005). Presence of the ER in dendritic spines varies between different neuronal cell types. Almost every single spine of cerebellar Purkinje neurons contains a tubule of smooth ER associated with the spinous actin cytoskeleton (Wagner et al., 2011a). In contrast, the majority of dendritic spines of pyramidal neurons in the cortex and hippocampus do not contain ER (Toresson and Grant, 2005). However, in those cells a subset of spines contain a complex ER-based organelle called the spine apparatus (SA; Deller et al., 2003). The SA is usually localized in the spine neck or at the base of the spine head and consists of laminar ER stacks with intervening electron-dense plates, connected to the main ER network. This intriguing structure serves as a Ca^2+^ store and is important for synaptic plasticity (Deller et al., 2003).

The protein synaptopodin is an essential component of the SA structure, since dendritic spines of synaptopodin-deficient mice can contain single ER tubules but are devoid of the SA, and expression of synaptopodin is sufficient to rescue this phenotype (Deller et al., 2003, 2007; Vlachos et al., 2013). Accordingly, cerebellar Purkinje neurons, which do not express synaptopodin, do not form the SA despite having ER tubules in all their numerous spines. Of note, synaptopodin is also required for the establishment of a similar ER-based structure, called the cisternal organelle, in the axon initial segment (AIS; Bas Orth et al., 2007). Expression of synaptopodin, which is restricted to renal podocytes and telencephalic neurons in the brain, is developmentally regulated and coincides with synaptic maturation (Czarnecki et al., 2005; Mundel et al., 1997). Synaptopodin is a cytosolic protein. It lacks a clear domain organization, contains several predicted disordered regions, and is known as an actin- and α-actinin-binding protein (Asanuma et al., 2005; Chalovich and Schroeter, 2010; Kremerskothen et al., 2005). It has been demonstrated that ∼20–30% of dendritic spines are positive for synaptopodin, and that it is more frequently present in a subset of spines with a large spine head volume (Vlachos et al., 2009; Holbro et al., 2009). Interestingly, the presence of synaptopodin correlates with synaptic strength, indicating that SA-containing spines might have different plastic properties (Korkotian et al., 2014; Vlachos et al., 2009). Along the same lines, the presence of a SA has been shown to regulate the diffusion of metabotropic mGluR5 glutamate receptors in and out of spines (Wang et al., 2016). Accordingly, synaptopodin^−/−^ mice show impaired long-term potentiation (LTP) and spatial learning (Deller et al., 2003; Korkotian et al., 2014).

Notably, synaptopodin mRNA and protein levels are regulated by neuronal activity. For example, it has been shown that in dentate granule cells synaptopodin expression is upregulated following LTP *in vivo* (Yamazaki et al., 2001). Moreover, a recent study has found that synaptopodin is required for cAMP-mediated LTP in developing neurons and that it is most likely a substrate of protein kinase A (PKA), which becomes activated during LTP (Zhang et al., 2013). Taken together, these data suggest that synaptopodin acts as a powerful tool to induce formation of the SA in dendritic spines, and it is very likely that synaptopodin expression, localization, function and stability are highly regulated.

Despite the importance of the SA in synaptic function, there are still many open questions about the origin of this organelle. For instance, it is unclear how synaptopodin and the SA are localized to a selected subset of dendritic spines. Is the complete SA actively transported along the dendrite and then targeted to selected spines, or it is assembled locally as needed? What are the molecular mechanisms that regulate SA localization?

In this study, we aimed to address these questions and to learn more about the dynamics of the spinous ER and synaptopodin in hippocampal neurons. In order to identify factors that allow for synaptopodin localization at postsynaptic sites, we performed a mass spectrometric analysis of brain-specific binding partners isolated via a pulldown assay. Interestingly, several myosins stood out as potential binding partners, including the processive motors myosin V (herein referring to both the myosin Va and Vb forms) and VI. While doing long-term live-cell imaging of primary neurons transfected with GFP–synaptopodin, we found no evidence of synaptopodin clusters being actively transported along dendritic branches. Instead, we observed gradual formation of clusters in spines, which we interpret as the SA being assembled on-site. Through analyzing myosin Va and myosin VI dominant-negative approaches, pharmacology and myosin VI-deficient mice, we show that myosin VI is dispensable for the spine localization of synaptopodin, whereas myosin V affected the formation and/or maintenance of synaptopodin clusters, resulting in diminished synaptic targeting of synaptopodin and the SA.

## RESULTS

### Processive myosins are novel interactors of synaptopodin

**Fig. 1.**
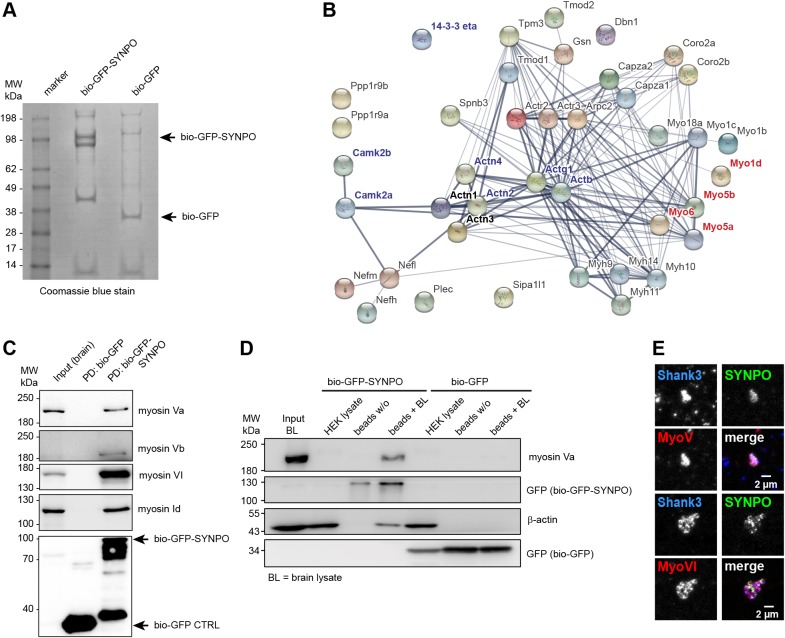
**Myosin family proteins are novel interaction partners of synaptopodin.** (A) SDS-polyacrylamide gel of the bio-GFP–synaptopodin (SYNPO) and bio-GFP (control) pulldown used for mass spectrometric analysis. Biotinylated GFP–synaptopodin was expressed in HEK293 cells and bound to Streptavidin beads. The beads were then incubated with rat brain lysate to pulldown brain-specific interactors of synaptopodin. (B) Network analysis of selected potential synaptopodin interactors identified in the mass spectrometry analysis (Table S1) using the online STRING analysis tool. Highlighted in blue are known synaptopodin-interacting proteins including actin (Actg1, Actb) and actinins (Actn2, Actn4). In red are myosins that were later tested positively in the co-immunoprecipitation shown in D. Line thickness indicates strength of data support. (C) Western blot analysis of bio-GFP–synaptopodin pulldown from brain lysate confirms interaction with myosin Va, Vb, VI and Id. Input, brain lysate; Strept-HRP, streptavidin coupled to horseradish peroxidase. Arrows indicate the expected molecular mass of bio-GFP–synaptopodin (upper) and bio-GFP (lower). (D) Western blot analysis of bio-GFP–synaptopodin pulldown probed for the presence of actin before and after incubation with brain lysate. Bio-GFP–synaptopodin-coupled beads are not enriched with myosin Va or actin coming from the HEK293 cells, but show association with myosin Va and actin specifically from brain lysate. BL, brain lysate. Beads w/o, bio-GFP–synaptopodin-coupled beads before incubation in brain lysate. Beads+BL, bio-GFP–synaptopodin-coupled beads after incubation in brain lysate. (E) Immunostaining of synaptosome-enriched fraction from rat brain. Synaptosomes were enriched from brain lysate using differential centrifugation and stained with antibodies against Shank3 (post-synaptic marker), synaptopodin and myosin V or VI. Both myosin V and VI could are found at synaptopodin- and Shank3-containing structures. Scale bar: 2 µm.

In humans and rodents, three splice isoforms of synaptopodin have been identified, but only the shortest isoform is found in the brain (Asanuma et al., 2005; Chalovich and Schroeter, 2010; Fig. S1). Previously, an interaction between the long splice isoform of human synaptopodin (UniProt identifier Q8N3V7-2; 903 amino acids) and α-actinin-2 has been suggested to mediate localization of (overexpressed) synaptopodin at spines via a binding motif at the C-terminus (Kremerskothen et al., 2005; Fig. S1). However several other studies have reported that the short splice isoform of mouse synaptopodin, lacking the proposed targeting domain, is also clearly enriched at spines (Fig. S1; Korkotian et al., 2014; Vlachos et al., 2009). Asanuma et al. showed that this short splice isoform contains two α-actinin-2- and α-actinin-4-binding sites of its own, but they did not study synaptopodin in brain (Asanuma et al., 2005). α-Actinin is non-selectively enriched in all types of spines, thus its role in localizing synpatopodin and the SA to selected spines is questionable (Hodges et al., 2014; Matt et al., 2018; Nakagawa et al., 2004). In order to identify the molecular mechanisms that govern the distribution of synaptopodin, we set out to first obtain unbiased information about the brain-specific synaptopodin interactome (the 690-amino-acid isoform; Fig. S1). Through a mass spectrometric analysis of a pulldown fraction from rat hippocampus with biotinylated synaptopodin produced in HEK293T cells (Fig. 1A,B), we found several known (including actinins, actin and 14-3-3 proteins) and many novel putative synaptopodin interaction partners (Fig. 1B; Tables S1, S2). Interestingly, the CamKIIα and CamKIIβ isoforms were also present in complex with synaptopodin, an interaction previously only known from renal podocytes (Faul et al., 2008). Newly identified proteins include several actin-stabilizing, -capping, -severing and -modifying proteins including tropomodulins (Tmod2, Tmod3), gelsolin (Gsn), Arp2/3 complex members (Arpc2), coronins (Coro2a and Coro2b) and F-actin-capping proteins (Capza1 and Capza2) (Fig. 1B; Tables S1, S2). This finding indicates that the association of synaptopodin and actin filaments might be more complex than just direct binding to actin and can be subjected to regulation. While we found no kinesin or dynein motor proteins, nor their adaptors present in the synaptopodin complex (Table S1), the fact that there were unconventional myosins as putative prominent binding partners attracted our attention (Fig. 1B). Processive myosin V and VI are highly expressed in pyramidal neurons. They mediate organelle trafficking in and out of dendritic spines and provide stable anchoring on actin filaments (Correia et al., 2008; Esteves da Silva et al., 2015). It has been shown that myosin Va is required for targeting of the ER into the spines of cerebellar Purkinje neurons (Wagner et al., 2011a). We hypothesized that the processive myosins V and VI could transport and anchor synaptopodin to dendritic spines, thereby mediating the synaptopodin-dependent formation of the spine apparatus. To verify these interactions, we performed a pulldown assay from rat hippocampus, and analyzed individual interactors via western blotting. This confirmed the association of bio-GFP–synaptopodin but not the bio-GFP control with the endogenous myosin Va, Vb, VI and Id (Fig. 1C). Interestingly, we found that β-actin was present in the synaptopodin–myosin Va complex formed in rat brain lysate, but not in the extract from HEK293T cells (Fig. 1D). Since all reactions were performed on ice and in the presence of detergents, the actin associated with the synaptopodin-beads must be either G-actin or a specific type of highly stabilized F-actin from the brain lysate. To further confirm the association of synaptopodin with processive myosins in the brain, we isolated a synaptosome-enriched fraction from rat cortex and hippocampus and immunostained it for endogenous synaptopodin, myosin Va, myosin VI and SHANK3, as a post-synaptic marker. This assay indicated that synaptopodin-positive synaptosomes indeed contain myosins (Fig. 1E). We therefore decided to investigate the role of these myosins in the synaptic targeting of synaptopodin.

### Synaptopodin is mainly present in spines containing ER and is upregulated during neuronal development

While synaptopodin labeling is frequently used as a marker for the SA (Deller et al., 2000, 2003), here we initially asked how the presence of ER in dendritic spines correlated with the presence of synaptopodin by using organotypic hippocampal slice cultures. Two-photon imaging of CA1 neurons electroporated with plasmids encoding tdimer2 as a morphology marker and ER–GFP as a label for the ER followed by post-hoc fixation and immunolabeling with a synaptopodin-specific antibody indicated that 71% of spines containing ER were also synaptopodin positive, and only 5% of spines without ER contained synaptopodin (Fig. S2A–C); therefore, we conclude that these spines contain a SA.

**Fig. 2.**
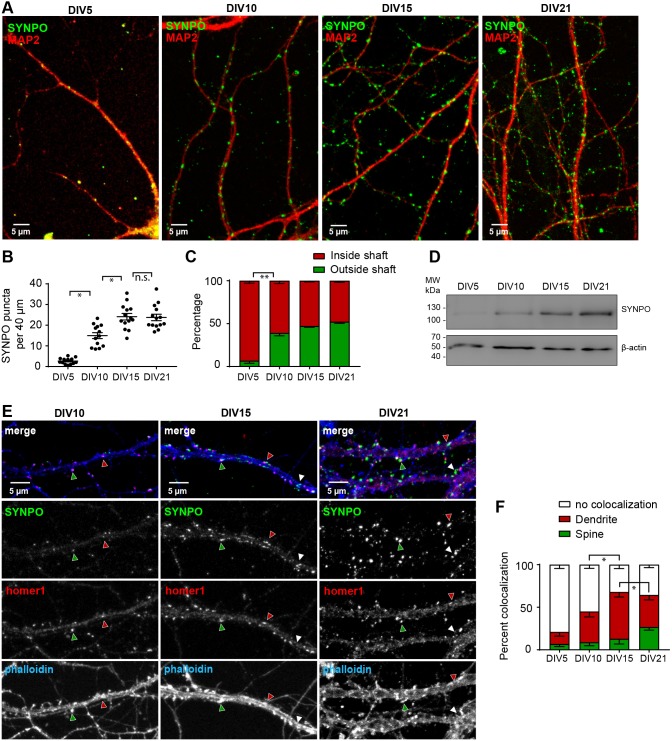
**Expression and localization of synaptopodin during development in hippocampal primary neurons.** (A) Representative confocal images of primary hippocampal neurons on DIV5, DIV10, DIV15 and DIV21 stained with anti-synaptopodin (SYNPO) and anti-MAP2 antibodies. Scale bars: 5 µm. (B) Quantification (mean±s.e.m.) of the average number of total synaptopodin puncta per 40 µm dendritic segments at DIV5, DIV10, DIV15 and DIV21. Kruskal–Wallis-Test with Dunnett's post hoc test *P=0.0493 (DIV5 vs DIV10), *P*=0.0421 (DIV10 vs DIV15); n.s., not significant. (C) Quantification (mean±s.e.m.) of synaptopodin puncta present inside or outside (spines, filopodia) of dendritic shafts. Kruskal–Wallis-Test with Dunnett's post hoc test **P=0.0077 (DIV5 vs DIV10). In B and C, DIV5, *n*=13 cells from three independent cultures with 44 dendritic segments counted; DIV10, *n*=14 cells from three independent cultures with 63 separate segments counted; DIV15, *n*=14 cells from two independent cultures with 93 separate segments counted; DIV21, *n*=16 cells from three independent cultures with 100 separate segments counted. (D) Immunoblot showing developmental expression of synaptopodin in primary hippocampal cultures. β-actin is used as loading control. (E) Representative confocal image of primary hippocampal neurons at DIV10, DIV15 and DIV21 stained with anti-synaptopodin, anti-homer1 and phalloidin–A647N. Red arrowheads, colocalization of synaptopodin and homer1 in dendrite; green arrowheads, colocalization in spine; White arrowheads, no colocalization. Scale bars: 5 µm. (F) Quantification (mean±s.e.m.) of the percentage of synaptopodin puncta colocalizing with homer1 in different dendritic subcompartments. Mixed ANOVA with DIV as between and localization as within group factors. *F*(4, 36)=4.4359, *P*=0.00512. Newman–Keuls post hoc test, *P=0.0348 (no coloc DIV10 vs DIV15), *P*=0.0435 (DIV15 vs DIV21 dendrite). DIV5, *n*=13 cells from three independent cultures with 44 separate segments counted; DIV10, *n*=8 cells from three independent cultures with 44 separate segments counted; DIV15, *n*=7 cells from two independent cultures with 35 separate segments counted; DIV21, *n*=8 cells from two independent cultures with 41 separate segments counted.

The expression of synaptopodin is developmentally regulated and coincides with synaptogenesis. This has been shown repeatedly in brain slices using antibody-staining against synaptopodin, as well as *in situ* hybridization to detect synaptopodin mRNA (Mundel et al., 1997; Czarnecki et al., 2005). However, no comprehensive timeline has been established for dissociated cultures of primary neurons. We therefore decided to analyze the expression of synaptopodin systematically and to establish a timeline of synaptopodin expression in hippocampal neurons prepared from embryonic day (E)18 rat embryos. Confocal imaging showed that at 5 days *in vitro* (DIV5) there were very few synaptopodin puncta found in dendrites labeled by MAP2 staining. At DIV10, a clear synaptopodin signal was detectable (Fig. 2A). The total number of puncta increased until DIV15 (Fig. 2B,C). Similarly, immunoblotting of cell lysates prepared from DIV5, 10, 15 and 21 primary hippocampal cultures indicated a gradual increase in synaptopodin expression during neuronal development and maturation (Fig. 2D). In the next set of experiments, we also stained for the postsynaptic marker homer1 and used the F-actin marker phalloidin, to visualize dendritic spines. We observed an increased colocalization of synaptopodin with homer1 in mature cultures (Fig. 2E,F). On average, ∼3% of synaptopodin puncta were adjacent to homer1-positive synapses in DIV5 neurons, while this number increased to 48%, 64% and 62% at DIV10, DIV15 and DIV21, respectively (Fig. 2F), indicating an increase in synaptic association of synaptopodin clusters. Interestingly, we observed a large number of synaptopodin clusters adjacent to homer1 in locations that seem to be inside the dendritic shaft, as opposed to spine localization. Some of those certainly can be accounted for by spines that reach out orthogonally to the plane of view. However, such spines are generally rare in primary neuronal culture, and we speculate that some of these sites might constitute excitatory shaft synapses that are associated with synaptopodin clusters, or even a proper SA structure. Taken together, these data show that synaptopodin expression increases during synaptogenesis and synaptic maturation in primary neuronal culture, and DIV15–21 neurons can be used as a cell culture model to study mechanisms regulating synaptopodin cluster localization and formation in dendritic spines.

### Synaptopodin clusters are closely associated with F-actin

**Fig. 3.**
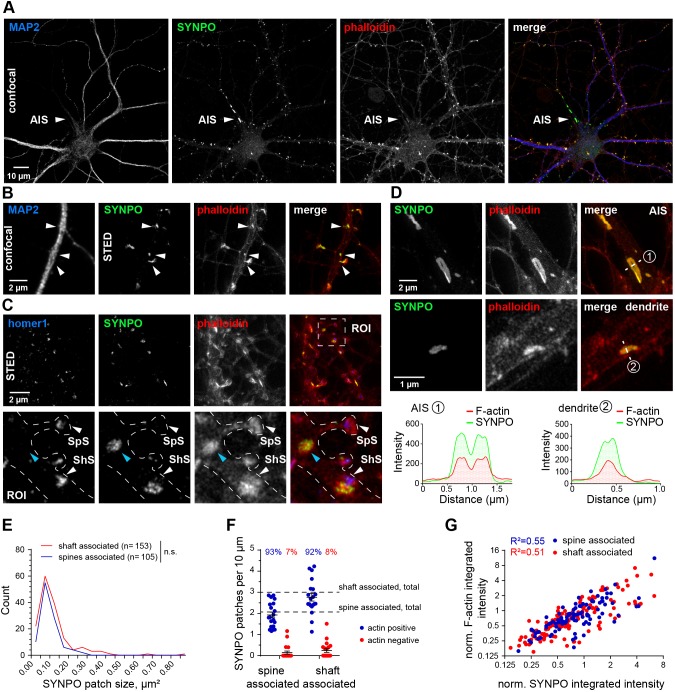
**Synaptopodin is closely associated with F-actin in spines and inside dendritic shafts.** (A) Confocal overview image of a DIV17 primary hippocampal neuron with F-actin stained by phalloidin–A647N and immunostaining of endogenous synaptopodin (SYNPO) and MAP2. White arrowheads indicate the AIS. Scale bar: 10 µm. (B) Confocal (MAP2) and STED image (phalloidin, synaptopodin) showing F-actin-enriched synaptopodin patches in spine necks and dendritic shafts. Scale bar: 2 µm. (C) Upper row, STED image of DIV17 hippocampal neuron stained for homer1, synaptopodin and F-actin (phalloidin). The box indicates the ROI shown in higher magnification in lower row. White arrowheads indicate homer1-positive synapses (SpS, spine synapse; ShS, shaft synapse). The shaft synapse is also associated with synaptopodin. Blue arrowheads indicate a dendritic synaptopodin cluster positive for F-actin but not associated with a synapse. Scale bar: 2 µm. (D) Comparison of the cisternal organelle found in the AIS (upper row) and dendritic synaptopodin patches (middle row). The lower row shows line profiles of phalloidin (F-actin) and synaptopodin intensity of the cisternal organelle (1) and dendritic synaptopodin patch (2). Scale bars: 2 µm (upper), 1 µm (middle). (E) Size distribution of synaptopodin patches associated with a synaptic spine or the dendritic shaft. Mann–Whitney *U*-test, *P*=0.35. Shaft-associated spots, *n*=153; spine-associated spots, *n*=105 from 20 dendritic segments of 17 neurons in two independent cultures. (F) Quantification of shaft- and spine-associated synaptopodin patches that are colocalizing with F-actin. *n*=20 dendritic segments of 17 neurons in two independent cultures. (G) Correlation of fluorescence intensity of synaptopodin and F-actin in shaft- and spine-associated synaptopodin patches. Shaft-associated patches: *n*=141; spine-associated patches, *n*=99; from 20 dendritic segments of 17 neurons in two independent cultures.

Synaptopodin is an actin-associated protein (Asanuma et al., 2005; Kremerskothen et al., 2005; Mundel et al., 1997). However, the spatial relation of synaptopodin puncta to neuronal F-actin has never been resolved in detail, owing to the diffraction limit of fluorescence imaging techniques used in earlier studies (Sánchez-Ponce et al., 2012; Vlachos et al., 2009). Only recently, super-resolution imaging based on stochastic optical reconstruction microscopy (STORM) and photoactivated localization microscopy (PALM) were used to show the association of overexpressed Dendra–synaptopodin with F-actin in the neck of dendritic spines in hippocampal primary neurons (Wang et al., 2016). Here, we decided to visualize endogenous synaptopodin by employing stimulated emission depletion (STED) nanoscopy. To this end, we stained hippocampal primary neurons with antibodies against synaptopodin and MAP2 or homer1, while the F-actin cytoskeleton was visualized by means of phalloidin–Atto647N. Two-color STED imaging of synaptopodin and F-actin indicated that synaptopodin-labeled structures were embedded inside an F-actin mesh both at the spine- and shaft localizations, as well as the AIS (Fig. 3A–D). As already shown in Fig. 2E,F, we again identified synaptopodin clusters close to homer1-positive synapses inside the dendritic shaft, which we hypothesize to constitute shaft-synapse-associated SAs. This leaves the question about the function of non-synapse-associated synaptopodin puncta inside the shaft. We suggest that, in analogy to the AIS, dendrites also may contain an equivalent of the cisternal organelle (Fig. 3C, blue arrow) that could be involved in dendritic Ca^2+^ regulation and buffering. In some cases, we were able to resolve a stack-like organization of individual synaptopodin clusters in dendrites, similar to those found in the AIS (Fig. 3D), which speaks in favor of this hypothesis. However, these occasions were rare since the stacks need to be perfectly orthogonal to the imaging plane to visualize them.

Comparative analysis of synaptopodin puncta showed that there were no significant differences in size distribution between spine-localized clusters and those in the shaft (Fig. 3E). In both cases more than 90% of them were enriched in F-actin (Fig. 3F) and there was a clear correlation between the intensity of synaptopodin and F-actin levels (Fig. 3G). Conversely, only 12% of the spine-associated clusters contained MAP2 (negative control, Fig. S3A). STED imaging revealed that homer1 signals were frequently adjacent to the synaptopodin clusters, but only in 17–24% of the cases was the signal directly overlapping (Fig. S3B). In a very recent study, we have shown that trafficking and localization of dendritic lysosomes is influenced by the presence of F-actin patches in dendrites, and that the long-term stalling of lysosomes is mediated by myosin V (Van Bommel et al., 2019). Therefore, we hypothesized that likewise, myosin V (or myosin VI) might play a role in dendritic targeting of synaptopodin, and decided to take a closer look at these motor proteins.

### Synaptopodin cluster numbers and their association with the ER in spines depend on myosin V

**Fig. 4.**
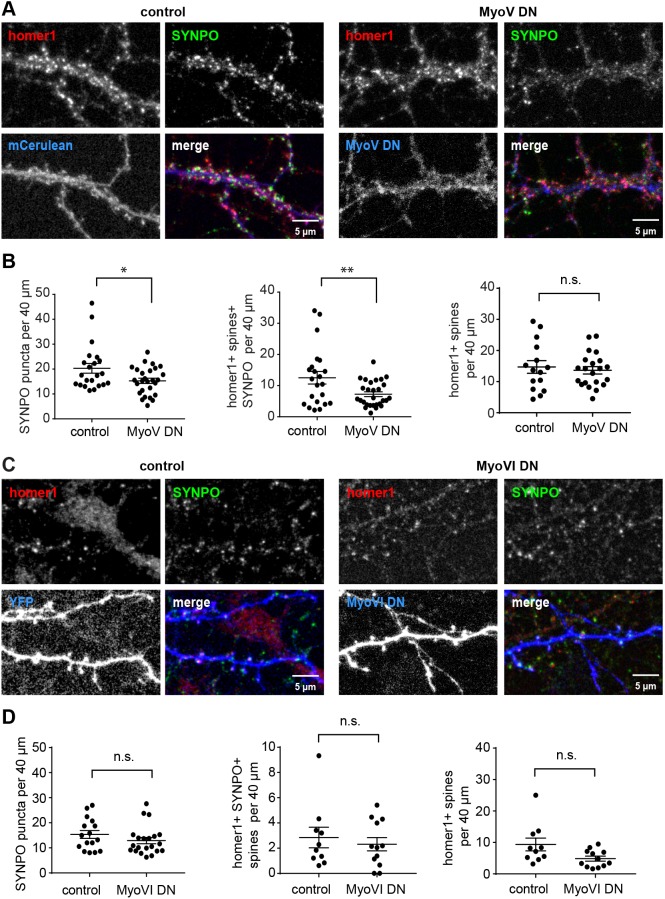
**Myosin V but not myosin VI inhibition affects spine localization of synaptopodin puncta.** (A) Representative confocal images of primary hippocampal neurons on DIV16 transfected with mCerulean (control) or Myosin-V-DN–mCerulean (MyoV DN) and stained with anti-synaptopodin (SYNPO) and anti-homer1 antibodies. Scale bar: 5 µm. (B) Quantification (mean±s.e.m.) of the number of total synaptopodin puncta (left), the number of homer1-positive spines containing synaptopodin (middle), and the total number of homer1-positive spines (right) per 40 µm dendritic segments in control and MyoV DN-expressing neurons. MyoV DN expression led to a decrease in total synaptopodin puncta (two-tailed unpaired *t*-test, *P=0.0218), and in puncta localizing to homer1-positive spines (two-tailed unpaired *t*-test, **P=0.0100), while the total number of spines was unchanged. MyoV DN, *n*=27 cells from four independent experiments with 51 segments counted. mCerulean, *n*=22 cells from four independent experiments with 54 segments counted. (C) Representative confocal image of primary hippocampal neurons on DIV16 transfected with mRuby2 (control) or GFP–Myosin-VI-dominant-negative (MyoVI DN) and stained with anti-synaptopodin and anti-MAP2 antibodies. Single-color channel and merged images are shown. Scale bar: 5 µm. (D) Quantification (mean±s.e.m.) of the number of total synaptopodin puncta (left), the number of homer1-positive spines containing synaptopodin (middle), and the total number of homer1-positive spines (right) per 40 µm dendritic segments in control and MyoVI DN-expressing neurons. Unpaired *t*-test (two-tailed) showed no significant change (ns). Left, Myo VI DN, *n*=21 cells from four independent experiments with 66 segments counted. Control, *n*=16 cells from four independent experiments with 40 segments counted. Middle and right, Myo VI DN, *n*=12 cells from two independent experiments with 21 segments counted. Control: *n*=10 cells from two independent experiments with 18 segments counted.

To test the contribution of myosin V and VI to the localization of synaptopodin clusters, we used a dominant-negative (DN) approach to inhibit the function of those myosins (Osterweil et al., 2005; Wu et al., 2002). The DN effect is achieved through overexpression of the C-terminal myosin cargo-binding domain, which competes with the endogenous myosin for cargo binding. Overexpression of the C-terminal myosin Va cargo-binding domain is well known to impair the function of endogenous myosin Va (Balasanyan and Arnold, 2014; Correia et al., 2008; Jones et al., 2000). Here, we developed a novel DN construct comprising only the globular tail domain of myosin Va, which dimerizes through a leucine zipper (LZ) in order to avoid additional effects mediated by the CC region that might sequester further interaction partners from their physiological targets. To verify the inhibitory effect of the myosin Va DN construct, we used Purkinje cells, since myosin Va is already known to mediate the targeting of ER into their dendritic spines (Fig. S3C). Confocal live imaging of primary mouse Purkinje cells showed that both constructs, that is with either the myosin Va CC or the LZ fused to the globular tail domain of the myosin, exert a DN effect on ER targeting to spines (Fig. S3C). Therefore, we decided to use the minimized LZ–globular domain fusion construct (hereafter referred to as MyoV DN) under the control of the human synapsin 1 promoter in hippocampal neurons. Similarly, the cargo-binding domain of myosin VI (MyoVI DN) fused to GFP and expressed under the synapsin promoter was used to inhibit myosin VI function (Van Bommel et al., 2019). We transfected DIV15–16 primary hippocampal neurons with DN constructs or corresponding controls, fixed them after 1 day of expression and stained for endogenous synaptopodin and homer1 (Fig. 4A–D). Both overexpressed DN constructs entirely filled dendrites and spines and thus could be used as a volume marker (Fig. 4A,C). MyoV DN expression resulted in a significant reduction of total synaptopodin clusters compared to control (on average 13.5 per 40 µm in MyoV DN and 24 per 40 µm in mCerulean control; Fig. 4B). Significantly less synaptopodin puncta were found in association with dendritic spines (Fig. 4B). Importantly, the total number of homer1-positive dendritic spines did not change (Fig. 4B). This indicates that myosin Va is required for the formation or stability of synaptopodin clusters and most likely the associated SA. Although quantification showed some reduction of synaptopodin puncta following overexpression of MyoVI DN, the difference to control neurons was not statistically significant (Fig. 4C,D). To corroborate these findings, we utilized primary hippocampal cultures from wild-type and myosin VI-deficient *Snell's waltzer* (*Myo6^sv/sv^*) mice (Avraham et al., 1995). Neurons were fixed at DIV8, 14 and 17 and stained for synaptopodin, homer1 and F-actin (Fig. S4A). Quantification of confocal images showed that, very similar to what was found for rat primary neuronal cultures, the number of synaptopodin puncta increased during development in both wild-type and *Myo6^sv/sv^* dendrites. Those numbers did not significantly differ between the genotypes (Fig. S4B). In addition, the distribution of homer1-colocalized synaptopodin clusters to spines and dendritic shafts was not statistically different in *Myo6^sv/sv^* neurons compared to wild-type (Fig. S4C). Thus, we conclude that the interaction of synaptopodin with myosin V, but not VI, is essential for the formation of synaptopodin clusters and for their normal distribution to dendritic spines.

**Fig. 5.**
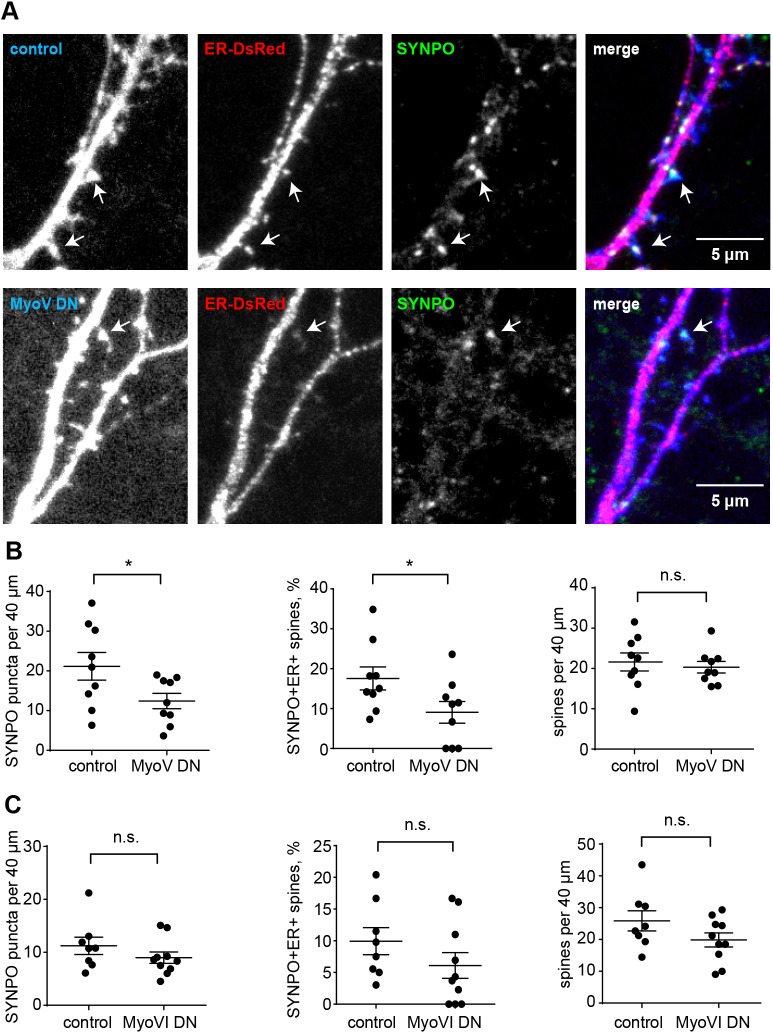
**Myosin V inhibition affects the number of spines containing both synaptopodin and the ER.** (A) Representative confocal images of primary hippocampal neurons on DIV17 transfected with an ER marker and a cell fill (YFP; control) or MyoV DN, stained with anti-synaptopodin (SYNPO) antibody. Arrows indicate dendritic spines that are positive for both synaptopodin and ER. Scale bars: 5 µm. (B) Quantification (mean±s.e.m.) of the number of synaptopodin puncta (left), the percentage of spines that are positive for both synaptopodin and ER (middle), and the number of spines per 40 µm (right). MyoV DN showed a decrease in total synaptopodin puncta (two-tailed unpaired *t*-test, *P=0.043) and in spines containing both synaptopodin and the ER (two-tailed unpaired *t*-test, *P=0.0481). Myo V DN, *n*=9 cells from two independent experiments with 22 segments counted. mCerulean, *n*=9 cells from two independent experiments with 23 segments counted. (C) Quantification (mean±s.e.m.) of the number of total synaptopodin puncta, the percentage of spines that are positive for both synaptopodin and ER, and the number of spines per 40 µm. Myo VI DN, *n*=10 cells from two independent experiments with 22 segments counted. Control, *n*=8 cells from two independent experiments with 22 segments counted. MyoVI DN had no significant effect on the measured parameters (ns).

Since the number of synaptically localized synaptopodin clusters was reduced upon inhibition of myosin V, next we asked whether this would also affect SA formation. To test this, we co-transfected primary neurons with an ER marker fused to DsRed and either MyoV DN, MyoVI DN or control plasmids, and fixed them after 1 day of expression. Since the DsRed signal proved to be too dim and prone to bleaching during imaging, in addition to the endogenous synaptopodin immunostaining, we stained the ER marker with an anti-RFP AbberiorStar-580 FluoTag (NanoTag). Confocal imaging showed that, similar to what was found in organotypic slices (Fig. S2), ER tubules could be seen inside a subset of dendritic spines (Fig. 5A). We interpreted colocalization of synaptopodin and the ER inside a dendritic spine as a SA. Quantitative analysis indicated that, as above, the total number of synaptopodin puncta was reduced upon overexpression of MyoV DN, but not MyoVI DN (Fig. 5B,C). The number of synaptopodin only- or the ER only-containing spines was slightly but not significantly decreased (Fig. S5A,B) but the number of spines containing both the ER marker and synaptopodin (SA) was reduced specifically after expression of MyoV DN (Fig. 5B), whereas the total spine density was again not affected by overexpression of either construct (Fig. 5B,C). From this, we conclude that inhibition of myosin V negatively affects the formation of the SA.

### Synaptopodin clusters do not exhibit processive trafficking, but show differences in their molecular dynamics depending on their localization

**Fig. 6.**
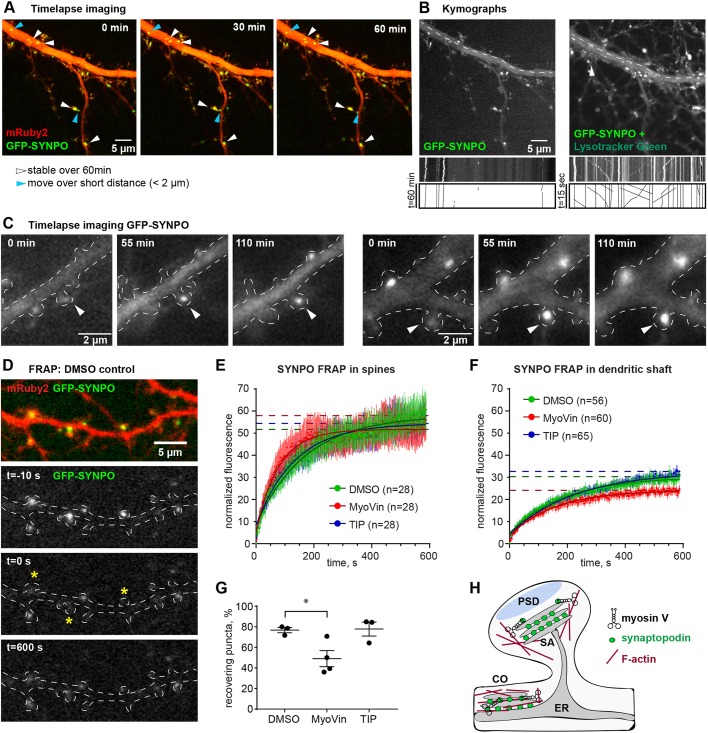
**Synaptopodin puncta are largely immobile and are generated *de novo* in dendritic spines, and synaptopodin turnover rate is affected by inhibition of myosin V, but not myosin VI.** (A) 60 min time-lapse imaging of a primary hippocampal neuron (DIV17) expressing GFP–synaptopodin (SYNPO) and mRuby2. Images were acquired at a 2 min interval. White arrowheads indicate synaptopodin puncta that were stably localized over the entire imaging period. Blue arrowheads indicate puncta that moved over very short distances (<2 µm). Scale bar: 5 µm. Also see Movie 1. (B) Shown is the same neuron as in A. Left, image and kymograph of the main dendritic shaft (dashed line) in the synaptopodin channel over the 60 min imaging period. Right: After 60 min imaging, Lysotracker Green was added to the imaging medium and the cell was imaged for 15 s with a 1 s interval. The kymograph shows moving lysosomes in the previously imaged cell. Scale bars: 5 µm. (C) Example images from a 110 min time-lapse imaging of a primary hippocampal neuron (DIV15, outlined with a dashed white line) expressing GFP–synaptopodin. Images were acquired with a 5 min interval. Synaptopodin puncta can be observed gradually emerging in dendritic spines (white arrowheads). Scale bars: 2 µm. Also see Movie 2. (D) Analysis of synaptopodin dynamics using FRAP. Example images of a DIV17 primary hippocampal neuron expressing GFP–synaptopodin and mRuby2, before, during and after photobleaching of selected synaptopodin puncta (asterisks). (E) FRAP quantification for synaptopodin puncta localized in spines. Compared were fluorescence recovery rates in control (DMSO) and myosin V-inhibitor (MyoVin, 30 µM for 20 min) or myosin VI-inhibitor (TIP, 4 µM for 20 min) treated cells. In the analysis, only those puncta that recovered >10% of their initial fluorescence were considered. One-phase-association fits, *n* numbers and plateaus are indicated (DMSO 58±1, MyoVin 52±0.7, TIP 54±0.8). (F) FRAP quantification for synaptopodin puncta localized in the dendritic shaft. One-phase-association fits, *n* numbers and plateaus are indicated (DMSO 30±0.4, MyoVin 24±0.3, TIP 33±0.5). For E and F, repeated measures two-way ANOVA showed treatment did not lead to a significant difference. (G) Percentage of puncta that were considered ‘recovering’ (recovered>10% of their initial fluorescence). One-way ANOVA with Dunnett's post hoc test for DMSO vs MyoVin *P=0.0376. DMSO, *n*=3 independent experiments with 84 puncta analyzed; MyoVin, *n*=4 with 124 puncta analyzed; TIP, *n*=3 with 81 puncta analyzed. (H) Model summarizing the role of myosin V and synaptopodin interaction in localization of the SA to actin filaments associated with spine or shaft synapses. PSD, post-synaptic density; SA, spine apparatus; CO, cisternal organelle.

As shown previously, the distribution of overexpressed GFP–synaptopodin closely matches the distribution of the endogenous protein (Vlachos et al., 2009). Here, we generated a construct for GFP–synaptopodin expression under the human synapsin 1 promoter, which is frequently used for low-to-moderate neuron-specific expression of proteins in primary neurons and organotypic slices (Kügler et al., 2003; Mikhaylova et al., 2016). We asked whether GFP–synaptopodin clusters are actively transported within the dendrite, and how they are recruited into or removed from dendritic spines. However, time-lapse imaging of GFP–synaptopodin and mRuby2 co-expressing neurons did not reveal a single long-range transport event in dendrites over 1 hour of imaging with a 2 min interval (Fig. 6A). Synaptopodin puncta in both spines and dendritic shafts were stably anchored at the same locations and sometimes oscillated within areas of 1–2 µm (Fig. 6A). Subsequent staining of lysosomes in the same cell showed processive bidirectional transport, ensuring that dendritic cargo trafficking in the observed cell was not compromised in any way (Fig. 5B). Continued imaging of transfected neurons over a 7 h period also did not reveal any processive movement of GFP–synaptopodin clusters (Fig. S5A; Movie 1). These observations are in line with our mass spectrometry results, as we did not find any kinesin or dynein motor proteins nor their adaptors present in the synaptopodin complex (Table S1). Based on this, we rule out the possibility that clusters of synaptopodin might be actively transported via long-distance microtubule-dependent active transport. This leaves the scenario in which synaptopodin clusters form locally *de novo*, possibly via local mRNA translation or recruitment of soluble synaptopodin, and then build up the SA in place. Further analysis of live imaging data confirmed that there are indeed instances where the synaptopodin signal gradually accumulated in a spine (Fig. 6C; Movie 2).

So far, we found that both dendritic and synaptic synaptopodin clusters are closely associated with patches of F-actin and are stable at their locations over many hours (Fig. 3, Fig. 6A; Fig. S5). To further characterize spine- vs dendritic shaft-localized synaptopodin clusters, we performed fluorescence recovery after photobleaching (FRAP) experiments in neurons expressing GFP–synaptopodin and mRuby2 as a morphology marker (Fig. 6D–G). Interestingly, a subset of the photobleached synaptopodin clusters did not recover any fluorescence at all, so only those clusters that recovered at least 10% of their initial fluorescence value were considered in the FRAP analysis. We found that the recovery rate of spine-localized puncta was twice as fast as in the shaft (Fig. 6E,F). Incubation of neurons with a pharmacological myosin V inhibitor, MyoVin, which blocks the ATPase activity of the motor domains and can thus turn myosin V from a processive motor into a tether (Gramlich and Klyachko, 2017; Heissler et al., 2017), or with myosin VI inhibitor (TIP) did not significantly affect the recovery kinetics at either location. However, the number of recovering synaptopodin puncta was significantly reduced upon treatment with MyoVin (Fig. 6E–G), which indicates an involvement of the motor in the local availability of synaptopodin.

Taken together, it is very likely that synaptopodin and the ER are locally emerging at synaptic sites to form a SA and myosin V, rather than myosin VI, is involved in the formation or stabilization of synaptopodin clusters and/or the SA, and possibly targeting to specific subcellular sites.

## DISCUSSION

Functional and structural properties of dendritic spines containing the SA differ from their neighbors without the SA, as it plays a key role in synaptic Ca^2+^ homeostasis (Korkotian et al., 2014; Vlachos et al., 2009). An essential component, the protein synaptopodin, is closely associated with the smooth ER-derived membranes that are forming the SA. Despite a growing number of studies addressing the role of the SA in neuronal function, it was still unclear how synaptopodin or the SA are localized to dendritic spines. In previous studies, long-term imaging experiments with overexpressed GFP–synaptopodin in primary hippocampal neurons revealed that synaptopodin clusters change their position within dendrites or spines only over large time spans (Vlachos et al., 2009.). However, since these data were acquired with a 1-day interval, it is difficult to judge whether synaptopodin clusters were actively relocated as a whole, or disassembled and newly assembled elsewhere. In this study, we aimed to characterize molecular dynamics, transport and synaptic targeting of synaptopodin in more detail.

Imaging of dendritic spines from CA1 hippocampal neurons in slice cultures indicated that the majority of dendritic spines containing ER were also positive for synaptopodin. In addition, in primary hippocampal neurons synaptopodin labels the SA (Korkotian et al., 2014). Previous studies have described a developmental regulation of synaptopodin expression, by using western blots of whole tissue-lysates and staining of hippocampal slices of post-natal rat pups (Mundel et al., 1997; Czarnecki et al., 2005). Here, we established a detailed timeline of synaptopodin expression and synaptic localization in cultured primary hippocampal neurons. We found that synaptopodin levels and the degree of synaptic localization increased with the maturation of the cultures and reached a maximum between DIV15 and DIV21. We therefore continued to use DIV17–DIV21 cultures for our experiments. Notably, this development is in accordance with data published on the synaptopodin-dependent maturation of the cisternal organelle of the AIS (Sánchez-Ponce et al., 2011, 2012). Interestingly, we discovered that synaptopodin clusters are not only found in dendritic spines and the AIS but are also present along the dendritic shaft. Both spine- and shaft-associated clusters were closely associated with F-actin, suggesting that not all shaft clusters were located in proximity to a synapse. It is therefore tempting to speculate that those, in contrast to SAs, could represent an ER-associated structure like the cisternal organelle, which is found at the AIS. Electron microscopy studies combined with immunogold labeling could shed some light on the identity of these structures in the future.

Until now there was a limited number of binding partners known for synaptopodin. Among them were the actin cross-linking α-actinin proteins (Asanuma et al., 2005). Some of the α-actinin isoforms are expressed in neurons and highly enriched in dendritic spines (Hodges et al., 2014; Nakagawa et al., 2004). However, the ubiquitous presence of α-actinins makes them unlikely to confer specific targeting of synaptopodin to selected dendritic spines. Taking this into account, we decided to search for new binding partners of synaptopodin that could shed some light on the biology of the spine apparatus. Using eukaryotically produced synaptopodin as bait we analyzed the brain-specific interactome of synaptopodin by mass spectrometry. In agreement with our live imaging data, there were no microtubule-based motor proteins or their adaptor proteins found in the synaptopodin complex. On the other hand, we detected a number of unique peptides corresponding to CaMKIIα, CaMKIIβ and 14-3-3 proteins. In renal podocytes, it has been shown that synaptopodin can be phosphorylated by PKA and CaMKII. Together with the phosphatase calcineurin, these kinases regulate the phosphorylation status of synaptopodin and its association with the protein 14-3-3, which can protect synaptopodin from degradation (Faul et al., 2008). Therefore, it is plausible that mechanisms similar to those in podocytes also regulate synaptopodin stability and degradation in dendritic spines. The most interesting and prominent interaction partners identified in this screen were several F-actin-based myosin motors. Processive myosin V and VI play an important role in a wide range of neuron-specific functions including organelle and mRNA transport and anchoring (Correia et al., 2008; Esteves da Silva et al., 2015). Class V myosins represented by myosin Va and Vb mediate spinous transport of membrane organelles, such as recycling endosomes, tubules of smooth ER or lysosomes (Wagner et al., 2011a; Wang et al., 2008; Van Bommel et al., 2019). Moreover, myosin V processivity is regulated by Ca^2+^ and calmodulin, which suggests that myosin function could be controlled by synaptic activity. Myosin VI is implicated in spinous vesicle trafficking as well, for example in Rab11 recycling endosomes (Esteves da Silva et al., 2015). These features make myosins very attractive candidates for synaptic targeting of synaptopodin. Using dominant-negative constructs, myosin VI-deficient mice and pharmacological inhibitors of myosin V and VI we found that interference with myosin V but not myosin VI reduced the number of synaptopodin clusters, as well as spinous targeting of synaptopodin and of the spine apparatus.

Interestingly, when we performed time-lapse imaging of GFP–synaptopodin-transfected neurons over extended periods of time, we observed only very short-distance movements (<2 µm) of synaptopodin puncta, and no long-distance trafficking events. This indicates that under basal conditions, synaptopodin clusters are stably localized and immobile. On the other hand, we observed that new synaptopodin clusters emerged on-site in dendritic spines and persisted over long time periods. Interestingly, GFP–synaptopodin clusters in dendritic spines and in the shaft had very different molecular dynamics: after photobleaching, spine-associated clusters recovered fluorescence intensity much faster than shaft-associated clusters, indicating that spine-localized clusters contain a larger dynamic pool of synaptopodin. Since both shaft- and spine-localized synaptopodin clusters are associated with the F-actin cytoskeleton, and the turnover rate of F-actin is the same in both locations (Van Bommel et al., 2019), it is unlikely that actin dynamics play a role in synaptopodin stability. One possible explanation could be that spine-associated synaptopodin clusters represent the SA, whereas shaft-associated clusters might constitute a different kind of ER-based compartment, possibly more similar to cisternal organelles. In this case, the differences in synaptopodin stability are due to the different nature and molecular composition of those organelles. Furthermore, we found that interaction with myosin V is important for recruitment and clustering of synaptopodin molecules at F-actin-rich areas. It is plausible that synaptic activity has an influence on synaptopodin accumulation, for example, via control of myosin V activity, or via downstream phosphorylation of synaptopodin. This would provide an additional explanation for the differences observed in spine- and shaft-localized synaptopodin dynamics.

As for the involvement of myosin V, there are several possible scenarios as to how it could contribute to SA formation. We speculate that, since myosin V is responsible for targeting the ER to synaptic spines in Purkinje neurons, it might also fulfil this role in hippocampal neurons. It is conceivable that myosin V is required to initially localize an ER protrusion to a dendritic spine, which would be followed by accumulation of synaptopodin and formation of the SA (Fig. 6H). Alternatively, spinous accumulation of synaptopodin clusters via myosin V could be the necessary first step in localized SA formation, followed by the capture of a transient ER protrusion. This clustering could be achieved via interaction of synaptopodin with actin-bound myosin V, or via anchoring of dendritic synaptopodin mRNA that is then locally translated (Balasanyan and Arnold, 2014). In our experiments, we could not discriminate between these scenarios. Considering that the activity of myosin V is Ca^2+^ dependent, in future studies it would be interesting to test whether the SA relocates in response to synaptic activity, as this could provide selectivity for synaptic targeting.

Taken all together, the results of this study extend our understanding on how synaptopodin and presumably the SA are targeted to the synapses via association with myosin V and actin filaments.

## MATERIALS AND METHODS

### Constructs

To produce the pEGFP-bio-synaptopodin construct, synaptopodin (mouse isoform 3, UniProt identifier Q8CC35-3) was subcloned from a pEGFP-Synaptopodin plasmid (Asanuma et al., 2005) into pEGFP-C1-bio (a kind gift from Anna Akhmanova, Utrecht University, Utrecht, The Netherlands) with SacII and SalI restriction. For cloning of the hSyn1–GFP–synaptopodin construct, GFP–synaptopodin was amplified via PCR, and pAAV-hSyn1-mRuby2 was digested with EcoR1 and HindIII to remove mRuby2. GFP–synaptopodin was inserted into the digested plasmid via homologous recombination (Jacobus and Gross, 2015). To produce the MyoVI DN construct, the Syn–GFP–synaptopodin vector was digested with HindIII and NdeI to remove synaptopodin, and the C-terminal domain of mouse myosin VI (bp 3177-3789; NCBI reference sequence: NM_001039546.2) was amplified via PCR and cloned into the backbone (Aschenbrenner et al., 2003).

Purkinje-specific expression plasmids pL7, pL7-mCER, pL7-mRFP-ER-IRES-GFP were described previously (Wagner et al., 2011b; 2011a). Plasmid pL7-mCER-Myo5a-CC-GTD corresponds to pL7-mCER containing a cDNA encoding the C-terminal part of mouse MYO5A (starting at residue 1194 of the brain-spliced isoform, transcript variant X6; NCBI reference sequence XM_006510832.3) inserted in frame at the 3′-end of the mCerulean-coding sequence. Similarly, pL7-mCER-Myo5a-GTD carries a cDNA encoding the globular tail domain of mouse MYO5A (starting at residue 1415, numbering according to brain-spliced isoform). Plasmid pL7-mCER-LZ was generated by inserting a sequence encoding the leucine zipper of GCN4 (MKQLEDKVEELLSK NYHLENEVARLKKLVGE) in frame at the 3′-end of the mCerulean-coding sequence. To generate pL7-mCER-LZ-Myo5a-GTD, a sequence encoding the globular tail domain of mouse MYO5A (starting at residue 1415, numbering according to brain-spliced isoform) was inserted in frame at the 3′-end of the leucine zipper of pL7-mCER-LZ.

A complete list of expression constructs used in this study is provided in Table S3.

### Antibodies and pharmacological components

Details of antibodies used are provided in Table S4, and pharmacological components in Table S5.

### Animals

Wistar rats Crl:WI(Han) (Charles River) and Wistar Unilever HsdCpb:WU (Envigo) rats were used in this study. Pregnant rats were euthanized for primary hippocampal cultures (E18), post-natal day (P)0 mice were used for primary cultures, female P5–6 rat pups for organotypic slice cultures and adult female rats for biochemistry. Animal experiments were carried out in accordance with the European Communities Council Directive (20110/63/EU) and the Animal Welfare Law of the Federal Republic of Germany (Tierschutzgesetz der Bundersrepublik Deutschland, TierSchG) approved by the local authorities of the city-state Hamburg (Behörde für Gesundheit und Verbraucherschutz, Fachbereich Veterinärwessen, from 21.04.2015) and the animal care committee of the University Medical Center Hamburg-Eppendorf. The mice used in this study were bred and maintained in the animal facility of the Center of Molecular Neurobiology of Hamburg (ZMNH), Hamburg, Germany. *Snell's waltzer* mice carrying a spontaneously arisen ∼1.0 kb genomic deletion in *Myo6* were obtained from The Jackson Laboratory (B6×STOCK *Tyr^c-ch^ Bmp5^se^ +/+ Myo6^sv^*/J; stock no. 000578) and were repeatedly backcrossed to C57BL/6J to obtain *Myo6^sv/+^* mice carrying the *Snell's waltzer* allele but lacking the *Tyr^c-ch^* and *Bmp5^se^* alleles (Avraham et al., 1995).

### Organotypic hippocampal cultures, electroporation and two-photon microscopy

Hippocampal slice cultures were prepared using female rat pups (Wistar) at P5–6 as described previously (Gee et al., 2017). No antibiotics were used during the preparation or in the culture medium. At DIV 7–9, cultures were transfected with a 1:1.5 mixture of expression vectors encoding tdimer2 (Campbell et al., 2002) and ER–EGFP (Holbro et al., 2009) each driven by the synapsin-1 promoter, using a Helios gene gun (Bio-Rad). Imaging of organotypic hippocampal cultures was performed 2–3 weeks after gene transduction. *Z*-stacks of apical dendrites from CA1 expressing neurons were acquired using a custom-built two-photon imaging setup. It was based on an Olympus BX51WI microscope equipped with a LUMFLN 60×W 1.1 NA objective (Olympus), controlled by the open-source software package ScanImage (Pologruto et al., 2003) written in Matlab (Mathworks). A pulsed Ti:Sapphire laser (MaiTai DeepSee, Spectra Physics) controlled by an electro-optic modulator (350-80, Conoptics) was used to excite tdimer2 and ER–EGFP simultaneously at 980 nm. Emitted photons were collected through objective and oil-immersion condenser (1.4 NA, Olympus) with two pairs of photomultiplier tubes (PMTs, H7422P-40, Hamamatsu). 560 DXCR dichroic mirrors and 525/50 and 607/70 emission filters (Chroma Technology) were used to separate green and red fluorescence. Excitation light was blocked by short-pass filters (ET700SP-2P, Chroma). Slices were submerged in artificial cerebrospinal fluid (ACSF) containing (in mM): 127 NaCl, 2.5 KCl, 2 CaCl_2_, 1 MgCl_2_, 25 NaHCO_3_, 1.25 NaH_2_PO_4_ and 25 D-glucose (pH 7.4, ∼308 mOsm, saturated with 95% O_2_/5% CO_2_) at room temperature. After two-photon live imaging, slices were fixed overnight at 4°C in a 2% (w/v) PFA/sucrose mixture and used for post hoc staining with anti-synaptopodin antibody.

### Immunohistochemistry and imaging of organotypic slices

For immunostaining, organotypic slices were washed three times in PBS, followed by overnight permeabilization with 1% Triton X-100 at room temperature. In between each of the following steps, using PBS-based solutions, the slices were washed in PBS. For quenching, slices were submerged in glycine (50 mM) for 1 h. Afterwards, Image-iT Fx enhancer (Thermo Fisher Scientific) was used to avoid unspecific labelling. Slices were incubated with rabbit primary antibody against rat synaptopodin (Sigma-Aldrich #S9442, 1:1000) for 24 h at 4°C. Next, the slices were incubated with secondary antibody Alexa Fluor 488-conjugated goat-anti-rabbit-IgG (1:1000) for 12 h at 4°C. Finally, the slices were washed and mounted using the Prolong Antifade Kit (Thermo Fisher Scientific). Images were taken using an Olympus FV300 confocal microscope equipped with an UPLSAPO 60×1.35 NA objective (Schindelin et al., 2012).

### Subcellular fractionation and synaptosome enrichment

The whole brain of an adult, female rat was homogenized in cold homogenization buffer (0.32 M sucrose, 5 mM HEPES, pH 7.4) in a ratio 1 g of tissue to 15 ml of buffer, using a Dounce homogenizer at 900 rpm. All steps were performed on ice or at 4°C. The homogenate was centrifuged for 10 min at 1000 ***g***, the supernatant (S1) was removed and kept on ice. The pellet was re-suspended in homogenization buffer and homogenized again in a Dounce homogenizer. After a second centrifugation step for 10 min at 1000 ***g***, the supernatant (S1′) was combined with S1 and centrifuged for 15 min at 12,000 ***g***. The supernatant (S2) was removed, and the pellet was re-homogenized in homogenization buffer and centrifuged at 12,000 ***g*** for 20 min. The resulting pellet (P2′, enriched in synaptosomes and small vesicular organelles) was resuspended in homogenization buffer. For immunostaining, the sample was fixed by adding 2.7% Roti-Histofix (Carl Roth), 2.7% sucrose and incubated for 1 h at room temperature. Glass coverslips were coated with poly-L-lysine (Sigma-Aldrich) for 4 h at room temperature and washed five times with milliQ H_2_O. 10 µl droplets of the fixed microsome fraction were put on top of a parafilm surface, poly-L-lysine-coated coverslips were placed on the droplets face-down and incubated for 25 min at room temperature. Then the coverslips were flipped and washed three in PBS. Staining with antibodies against synaptopodin, shank3 and myosin V or VI was performed as described under in the immunocytochemistry section. The samples were imaged using a Leica TCS SP5 confocal microscope.

### Primary neuronal culture and transfections

Primary hippocampal rat cultures were essentially prepared as described previously (Kapitein et al., 2010). In brief, hippocampi were dissected from E18 embryos, treated with trypsin (0.25%, Thermo Fisher Scientific) for 10 min at 37°C, physically dissociated by pipetting through a syringe, and plated on 18 mm glass coverslips coated with poly-L-lysine (Sigma-Aldrich) at a density of 40,000–60,000 cells/ml and covered in DMEM (Gibco) with 10% fetal calf serum (FCS; Gibco) and penicillin/streptomycin (Thermo Fisher Scientific). After 1 h, the medium was replaced by BrainPhys neuronal medium supplemented with SM1 (Stem Cell # 5792) and 0.5 mM glutamine (Thermo Fisher Scientific) without antibiotics. Cells were then kept in an incubator at 37°C, 5% CO_2_ and 95% humidity. Primary hippocampal rat cultures were transfected with Lipofectamine 2000 (Thermo Fisher Scientific) according to the manufacturer's introductions and as described previously (Kapitein et al., 2010).

Primary hippocampal mouse cultures from *Myo6^sv/sv^* and *Myo6^+/+^* mice were prepared as described previously (Spilker et al., 2016). Briefly, hippocampi were dissected from male and female P0 *Myo6^sv/sv^* and *Myo6^+/+^* mice and cells were dissociated after 10 min treatment with trypsin at 37°C. Neurons were plated on glass dishes coated with poly-L-lysine (Sigma-Aldrich) at a density of 30,000 cells per well in a 12-well plate) in DMEM (Gibco) supplemented with 8% FCS, 1% penicillin/streptomycin. Following attachment, mouse cultures were kept in Neurobasal medium (Gibco) supplemented with 2 mM glutamine, 1% penicillin/streptomycin and 1× B27 supplement (Gibco), at 37°C, 5% CO_2_ and 95% humidity.

Dissociated cerebellar cultures containing Purkinje cells were prepared and transfected as described in detail previously (Wagner et al., 2011b).

### Western blot analysis of synaptopodin expression

At DIV5, DIV10, DIV15 and DIV21, cells were harvested by removing the medium and adding SDS sample buffer [250 mM Tris-HCl, pH 6.8, 8% (w/v) SDS, 40% (v/v) glycerol, 5% (v/v) β-mercaptoethanol and 0.004% Bromophenol Blue, pH 6.8) directly to the well. In an initial western blot against β-actin, the intensity of the actin band in each sample was measured with the gel analyzer/plot lanes function of ImageJ. For the final western blot, the inputs were normalized to contain the same amount of β-actin, as a loading control, and synaptopodin levels were detected using a synaptopodin antibody (Synaptic Systems #163002).

### Production of AAV and infection of primary hippocampal neurons

Adeno-associated viruses were produced at the Vector Facility of the University Medical Center Hamburg-Eppendorf (UKE). pAAV-hSyn1-mRuby2 (Addgene 99126; deposited by Viviana Gradinaru) and pAAV-hSyn1-GFP-synaptopodin (this study) were respectively packaged by pE2/rh10 and p5E/9 (Julie C. Johnston, University of Pennsylvania, USA) and pHelper (CellBiolabs). For infection with AAV, the viruses were added directly into the culture medium at final concentrations of between 10^9^ and 10^11^ vg/ml. pSyn-GFP-Synaptopodin AAV9 was added to the culture at DIV10, mRuby2 AAVrh10 was added on DIV14, and cells were imaged on DIV17.

### Immunocytochemistry

Cells were fixed in 4% Roti-Histofix (Carl Roth), 4% sucrose in PBS for 10 min at room temperature and washed three times with PBS, before they were permeabilized in 0.2% Triton X-100 in PBS for 10 min. After three washes in PBS, coverslips were incubated in blocking buffer (BB, 10% horse serum and 0.1% Triton in PBS) for 1 h at room temperature. Incubation with primary antibodies was performed in BB at 4°C overnight. After three washes in PBS, cells were incubated with corresponding secondary antibodies in BB for 1 h at room temperature. If the staining included phalloidin, an additional step was added where the coverslips were incubated with phalloidin-647N (1:40) overnight at 4°C. Finally, the coverslips were washed five times in PBS and mounted in mowiol.

### Confocal microscopy – fixed and live cell imaging

*Z*-stack images of fixed primary hippocampal neurons and fixed synaptosomal preparations were acquired on Leica TCS SP8 and Leica TCS SP5 confocal microscopes using a 63.0×1.40 NA oil objective using 488 nm, 568 nm and 633 nm excitation lasers. The pixel size was set to 90 nm and *Z*-steps varied between 250 and 350 nm. For the shown representative confocal images, a Gaussian filter (radius 0.5 px) was applied in ImageJ to reduce the visible background noise. Live Purkinje cells were imaged at 37°C on a Zeiss LSM 510 confocal microscope equipped with a 100×1.40 NA oil objective exactly as described previously (Wagner et al., 2011a,b).

### Analysis and quantification synaptopodin colocalization with homer1 or the ER

All of the following quantifications were undertaken from confocal Z-stack images of fixed and stained neurons. *Z*-stacks of dendrites were projected into one plane using the *Z* projection function of Fiji/ImageJ (Max Intensity). Per cell, between one and four dendritic segments of ∼40–60 µm length were selected and used for manual quantification as outlined below. The detailed information on the number of dendrites and cells is indicated in the corresponding figure legends.

For initial quantification of density and localization of synaptopodin puncta during neuronal development (Fig. 2A), untreated hippocampal neurons were fixed and stained against synaptopodin and MAP2 (a dendritic marker). The number of synaptopodin puncta that were localized inside the dendrite (as judged by MAP2 staining) or outside the dendrite (interpreted as filopodia or spine localization) were counted.

For quantification of synaptopodin localization with respect to developing synapses, untreated hippocampal neurons were fixed and stained against synaptopodin, homer1 (synaptic marker) and phalloidin (F-actin marker). Spines were defined as protrusions from the main dendrite that were positive for homer1.

To assess the effects of myosin V and myosin VI, DN or control constructs (mCerulean or YFP) were transfected on DIV16; cells were fixed on DIV17 and stained against synaptopodin and homer1 (Fig. 4; Fig. S4). Spines were defined as protrusions from the main dendrite that were positive for homer1.

Similarly, to quantify spinous localization of synaptopodin and the ER (Fig. 5), myosin DN or control constructs were co-transfected with an ER–DsRed marker on DIV16; cells were fixed on DIV17 and stained with anti-synaptopodin and anti-RFP. Here, spines were defined as spine-shaped protrusions from the main dendrite as visible in the cell fill.

### Fluorescence recovery after photobleaching

Primary hippocampal neurons were co-infected with two AAVs containing GFP–synaptopodin and mRuby2. mRuby2 was imaged with a 563 nm laser and the images were used to determine the localization of synaptopodin puncta. FRAP of GFP–synaptopodin was performed using a Nikon spinning disc confocal microscope. Time-lapse imaging of GFP–synaptopodin was performed with a 488 nm laser and bleaching of selected puncta was achieved with a 405 nm laser. Images were acquired at 0.5 Hz for 300 s, starting with a five frame baseline before FRAP. FRAP analysis was undertaken using the plug in Time Series Analyzer V3 of Fiji (NIH, Bethesda, MD, USA). Regions of interest (ROIs) were selected on the bleached (‘frapped’) synaptopodin puncta, on a non-bleached stretch of dendrite to account for bleaching during imaging (bleach control) and on a non-fluorescent region to account for background fluctuations. The integrated density values of those ROIs were measured, the background values were subtracted from the values of GFP–synaptopodin puncta, then GFP–synaptopodin values were normalized to the bleach control and to the pre-bleach value (i.e. the pre-beach value was considered as 100%).

### STED imaging

Confocal and STED images of phalloidin (Atto647N), MAP2 (Alexa Fluor 488) and synaptopodin (Abberior Star 580) were acquired on a Leica TCS SP8-3X gated STED microscope equipped with a pulsed 775 nm depletion laser and a pulsed white light laser (WLL) for excitation. For acquiring images, the Leica oil objective HC APO CS2 100×1.40 NA was used. Fluorescence of the respective channel was excited by the WLL at 650 nm (STED and confocal), 488 nm (confocal mode) and 561 nm (STED and confocal), respectively. For STED imaging, emission was acquired at 660–730 nm for Atto-647N and 580–620 nm for Abberior Star 580. The detector time gates for both STED channels were set from 0.5–1 ns to 6 ns. Both dyes were depleted with 775 nm. Images were taken as single planes of with 18 nm^2^ pixel size.

### Analysis of STED data

The number, size and integrated fluorescence intensity of synaptopodin patches, the presence of F-actin and association with homer1 or MAP2 were analyzed using Fiji. 2D STED images of dendritic stretches were used for analysis. ROIs around synaptopodin patches were drawn manually in the synaptopodin channel and, subsequently, integrated intensities within these ROIs were measured in the F-actin channel. Colocalization of synaptopodin with homer1 or MAP2 was scored manually by the presence or absence of a signal in the respective channels inside of the synaptopodin ROI. Differentiation between spine- and shaft-associated was based on the proximity to clearly discernable dendritic spines (not further than 0.5 µm from the spine base).

### Wide field and TIRF microscopy: live cell imaging

Live cell wide-field and TIRF imaging for GFP–synaptopodin motility analysis was performed on a Nikon Eclipse Ti-E controlled by VisiView software (VisitronSystems). The microscope was equipped with 488 nm, 561 nm and 640 nm excitation laser lines via a multi-mode fiber. Oblique and TIRF illuminations were achieved with an ILAS2 TIRF system. The angle of the excitation light was adjusted manually to achieve an optimal signal-to-noise ratio. Samples were imaged with a 100× TIRF objective (Nikon, ApoTIRF 100×/1.49 oil). Emission light was captured through a quad-band filter (Chroma, 405/488/561/640) followed by a filter wheel with filters for GFP (Chroma, 525/50 m), RFP (Chroma, 595/50 m), and Cy5 (Chroma, 700/75 m). Multi-channel images were acquired sequentially with an Orca flash 4.0LT CMOS camera (Hamamatsu).

### Culturing and transfection of HEK293 cells

HEK293T cells (as described in Mikhaylova et al., 2018) were maintained in full medium consisting of Dulbecco's modified Eagle's medium (DMEM; GIBCO, Thermo Fisher Scientific) supplemented with 10% fetal calf serum (FCS), 1×penicillin/streptomycin and 2 mM glutamine at 37°C, 5% CO_2_ and 95% humidity. For the expression of biotinylated proteins, HEK293T cells were grown in full medium made with a 50% DMEM, 50% Ham's F-10 Nutrient Mix (GIBCO, Thermo Fisher Scientific) mixture. Transfection was done using MaxPEI 25K (Polysciences) in a 3:1 MaxPEI:DNA ratio.

### Pulldown for western blot and mass spectrometry analysis

HEK293T cells were co-transfected with HA–BirA and either Synaptopodin–pEGFP-bio or pEGFP-bio (control vector) and harvested after 18 h of expression. The cells were lysed in extraction buffer [20 mM Tris-HCl pH 8, 150 mM NaCl, 1% Triton X-100, 5 mM MgCl_2_, complete protease inhibitor cocktail (Roche)], kept on ice for 30 min and centrifuged for 15 min at 14,000 ***g***. Magnetic Streptavidin M-280 Dynabeads (Invitrogen) were washed three times in washing buffer (20 mM Tris-HCl pH 8, 150 mM KCl, 0.1% Triton-X 100), blocked in 3% chicken egg albumin (Sigma-Adlrich) for 40 min at room temperature, and again washed three times in washing buffer. The cleared cell lysate was added to the blocked beads and incubated at 4°C on a rotator overnight. After the incubation period, the beads were washed twice with low-salt washing buffer (100 mM KCl), twice in high-salt washing buffer (500 mM KCl) and again twice in low-salt washing buffer. For preparation of whole rat brain extract, 9 ml lysis buffer [50 mM Tris-HCl pH 7.4, 150 mM NaCl, 0.1% SDS, 0.2% NP-40, complete protease inhibitor cocktail (Roche)] were added per 1 g of tissue weight, and the tissue was lysed using a Dounce homogenizer. The lysate was cleared first for 15 min at 1000 ***g***, and the supernatant was again centrifuged for 20 min at 15,000 ***g*** to obtain the final lysate. The washed beads were then combined with 1 ml of the cleared rat brain lysate and incubated for 1 h at 4°C on a rotator. Finally the beads were washed five times in washing buffer and resuspended in Bolt LDS sample buffer (Invitrogen) for subsequent SDS-PAGE. For mass spectrometric analysis, the samples were separated on a commercial Bolt Bis-Tris Plus Gel (Invitrogen) and the intact gel was sent to Erasmus MC Proteomics Center, Rotterdam, for mass spectrometry analysis (see below). For western blot analysis, the samples were loaded on 4–20% acrylamide gradient gels and blotted onto PVDF membranes. After blocking in 5% skim milk in Tris-buffered saline (TBS; 20 mM Tris-HCl pH 7.4, 150 mM NaCl and 0.1% Tween-20), membranes were incubated with primary antibodies diluted in TBS-A (TBS pH 7.4, 0.02% sodium azide) overnight at 4°C. Corresponding HRP-conjugated secondary antibodies and HRP–Strepdavidin were applied for 1.5 h at room temperature in 5% skim milk in TBS. The membranes were imaged on a ChemoCam imager (Intas).

### Mass spectrometry analysis

SDS-PAGE gel lanes were cut into 1-mm slices using an automatic gel slicer. Per sample, eight or nine slices were combined and subjected to in-gel reduction with dithiothreitol, alkylation with iodoacetamide and digestion with trypsin (Thermo Fisher Scientific; TPCK treated), essentially as described by Wilm et al. (1996). Nanoflow LCMS/MS was performed on an EASY-nLC 1000 Liquid Chromatograph (Thermo Fisher Scientific) coupled to an Orbitrap Fusion™ Tribrid™ Mass Spectrometer (Thermo Fisher Scientific) operating in positive mode and equipped with a nanospray source. Peptide mixtures were trapped on a nanoACQUITY UPLC C18 column (100 Å, 5 µm, 180 µm×20 mm, Waters). Peptide separation was performed on a ReproSil C18 reversed phase column (Dr Maisch GmbH; 20 cm×100 µm, packed in-house) using a linear gradient from 0 to 80% B [A, 0.1% formic acid; B, 80% (v/v) acetonitrile, 0.1% formic acid] in 60 min and at a constant flow rate of 500 nl/min. Mass spectra were acquired in continuum mode; fragmentation of the peptides was performed in data-dependent mode. Peak lists were automatically created from raw data files using the Mascot Distiller software (version 2.1; MatrixScience). The Mascot search algorithm (version 2.2, MatrixScience) was used for searching against a UniProt canonical database (release 2016_07, taxonomies Homo sapiens and Rattus norvegicus combined). The peptide tolerance was typically set to 10 ppm and the fragment ion tolerance to 0.8 Da. A maximum number of two missed cleavages by trypsin were allowed and carbamidomethylated cysteine and oxidized methionine were set as fixed and variable modifications, respectively. Quantitative analysis was peformed with MaxQuant software (version 1.5.4.1) using similar or standard settings and using the UniProt rat and human isoforms databases (both releases 2016_07).

### Network analysis of potential synaptopodin interactors

We selected the 50 proteins that had the highest unique peptide count in the synaptopodin pulldown (and where unique peptides were at least found at twice the level as in the negative control) from the mass spectrometry analysis of synaptopodin interactors (Table S1) for the network analysis using the ‘Multiple Proteins’ function of the online STRING database (http://string-db.org; Szklarczyk et al., 2015). The parameters changed from the default settings were as follows: Network edges: ‘Confidence’ (line thickness indicates the strength of data support). Active interaction sources: ‘Experiments’ and ‘Databases’. Selected network statistics/Functional enrichments: Biological Process (GO).

### Statistical analysis

Statistical analysis was performed in Statistica 13 (Dell Inc; RM two-way ANOVA and mixed ANOVA) or in Prism 6.05 (GraphPad; all other tests). Detailed information about the type of test used, significance levels, *n* numbers and biological replicates are provided in the figure legends. Experimental repeats and *n* numbers per experiment were chosen according to experience with effect size. All averaged data are shown as mean±s.e.m. The data were tested for normality using the D'Agostino-Pearson test (Prism) and accordingly subjected to a parametric (*t*-test or ANOVA) or non-parametric tests (Mann–Whitney test or Kruskal–Wallis test) for significance. The analysis of the *Myo6^sv/sv^* and *Myo6^+/+^* mice data was performed by researchers who were blind to the genotype of the mice.

## Supplementary Material

Supplementary information
