# Supplementary material for: Myosin V regulates synaptopodin clustering and localization in the dendrites of hippocampal neurons
Source: J Cell Sci. 2019 Aug 22;132(16):jcs230177. doi: 10.1242/jcs.230177 (PMC6737913; doi:10.1242/jcs.230177)
Supplement: Supplementary information [file joces-132-230177-s1.pdf]

SUPPLEMENT

SUPPLEMENTAL FIGURES

Figure S1

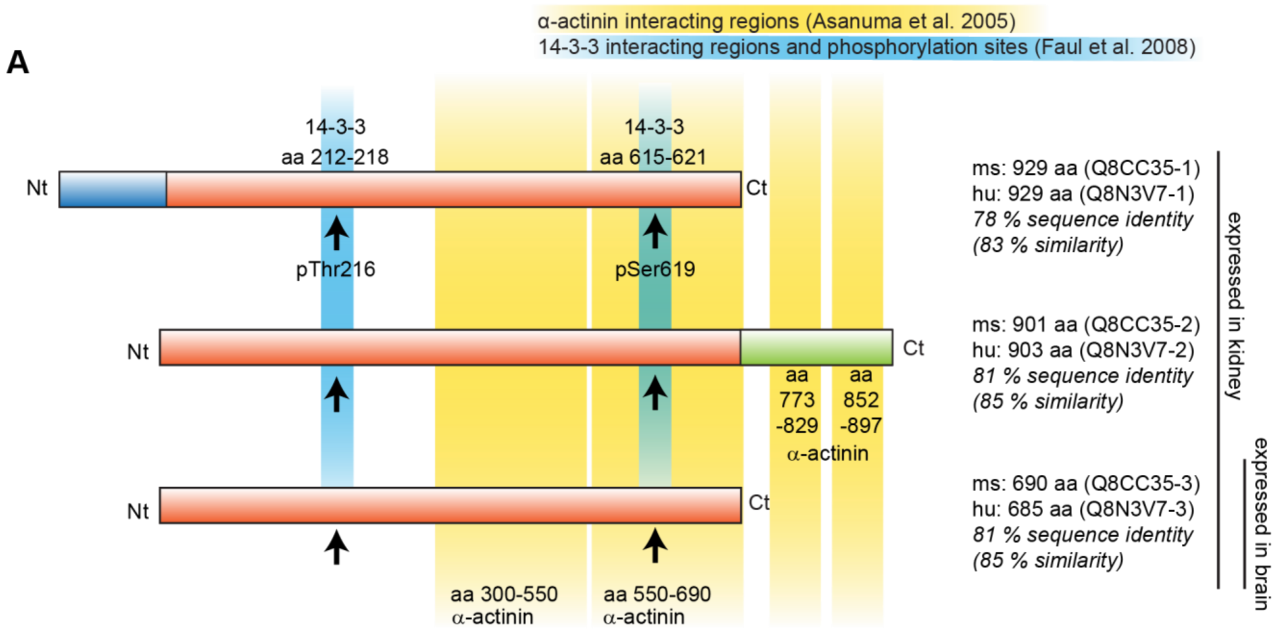

Figure S1. Synaptopodin splice isoforms

In mice, rats and humans, three splice isoforms of synaptopodin have been described. Isoform 1 has a unique N-terminus, and isoform 2 a unique C-terminus, while all of them share the same middle region. It was shown that only the shortest isoform 3, used in this study, is expressed in the brain. It contains two α-actinin binding sites, two 14-3-3 binding sites and two phosphorylation sites for CaMKII and PKA.

Journal of Cell Science • Supplementary information

Figure S2

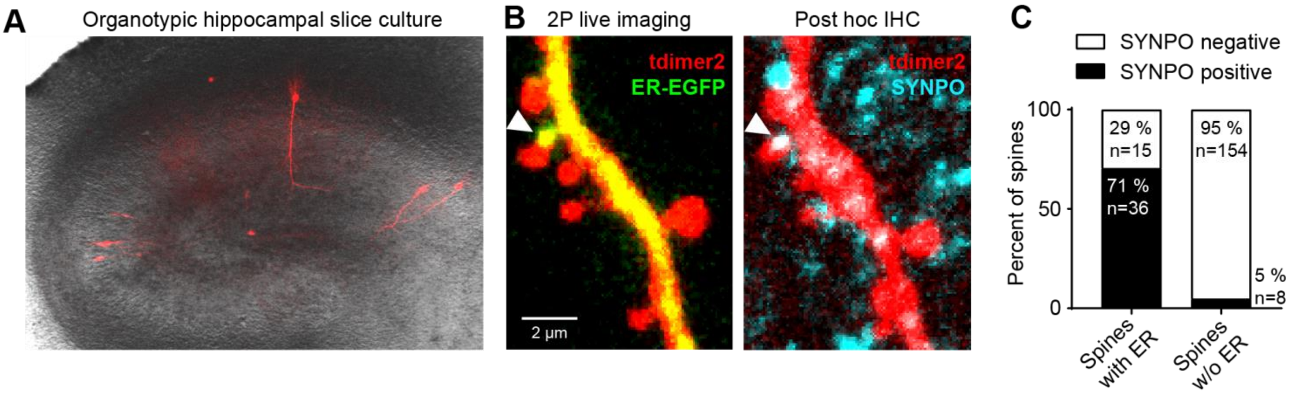

Figure S2. Synaptopodin co-localization with the ER in organotypic hippocampal slices

(A) Overlay of DIC and epifluorescence image of an organotypic hippocampal slice with few CA1 pyramidal neurons expressing tdimer2 and ER-EGFP.

(B) Two-photon live imaging from an apical dendritic segment of a CA1 neuron (left) and post hoc antibody staining against synaptopodin (right). Arrowhead shows a spine containing ER with positive staining for synaptopodin. Scale bar = 2 µm.

(C) Quantification of synaptopodin staining in spines according to presence or absence of ER in 2P live imaging (2 slices, 2 neurons).

Journal of Cell Science • Supplementary information

Figure S3

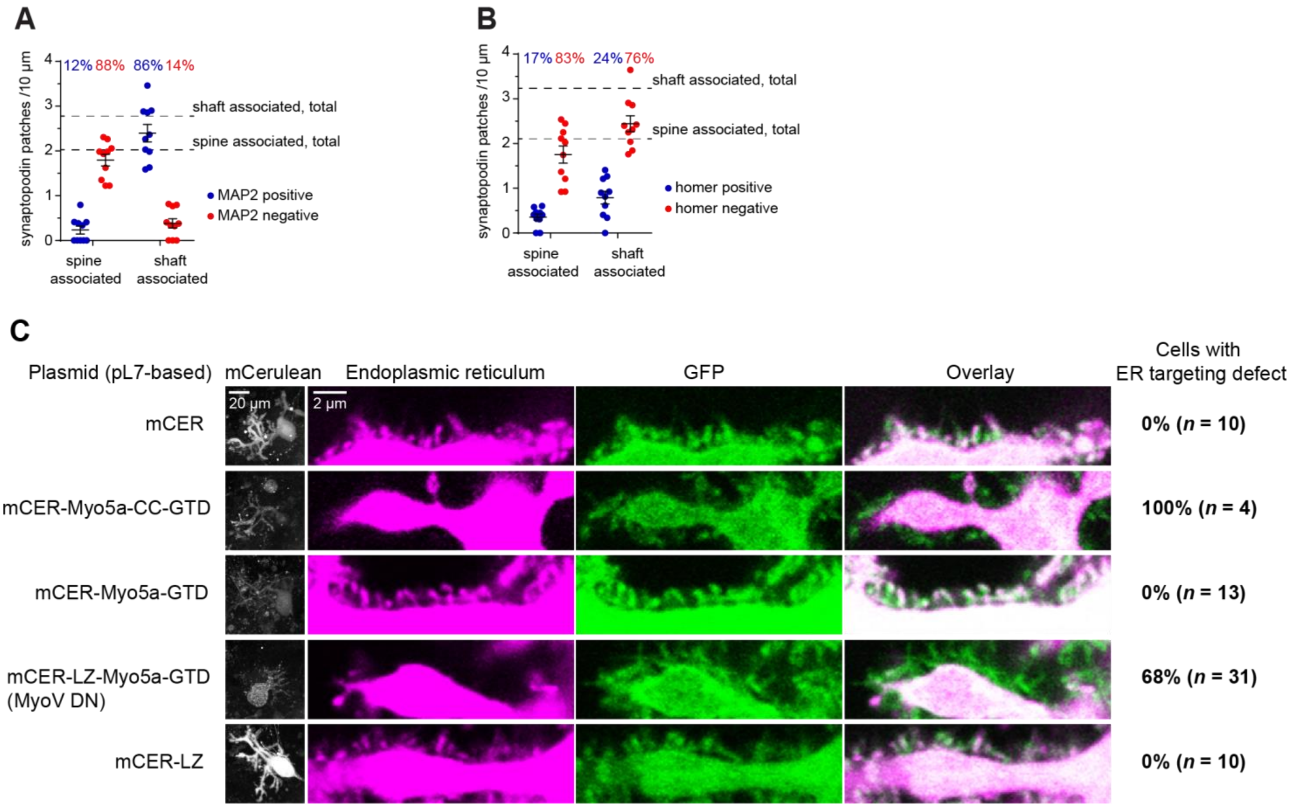

Figure S3. Controls of Figure 3 & Expression of a minimized myosin Va construct exerts a dominant-negative effect on ER targeting to dendritic spines of Purkinje cells

(A, B) Quantification of the co-localization of shaft- and spine-associated synaptopodin with MAP2 (A) and homer1 (B) from STED images shown in Fig. 3. (C) Representative confocal images of cultured Purkinje cells co-transfected with the indicated pL7 plasmid and with plasmid pL7-mRFP-ER-IRES-GFP to express both an ER marker (magenta) and a volume marker (*GFP*, green). Left images (*mCerulean*) depict the mCerulean signal in the somato-dendritic compartment to confirm transfection. Scale bar = 20 µm (*mCerulean* panels) and 2 µm (other panels). Based on ER- and GFP-images of dendrites, DIV13-15 Purkinje cells were scored for the presence of a *dilute-lethal*-like phenotype (i.e. less than 30% of spines contain ER). The percentage of cells displaying such an ER targeting deficit is depicted, *n* indicates the number of cells analyzed. No cells with a spine ER targeting deficit (0%) are present in the control situation (*mCER*; expression of unfused mCerulean) or upon expression of an mCerulean-tagged Myo5a globular tail domain without any coiled-coil sequence (*mCER-Myo5a-GTD*). Expression of a Myo5a fragment comprising half of the myosin's coiled-coil region plus the globular tail domain (*mCER-Myo5a-CC-GTD*) disrupts localization of ER to spines in 100% of examined cells. Expression of a similar construct in which the Myo5a coiled-coil is replaced by an established dimerization domain (the *GCN4* leucine zipper) also causes a spine ER targeting deficit (in 68% of examined cells; *mCER-LZ-Myo5a-GTD*). An mCerulean-leucine zipper fusion (*mCER-LZ*) does not disrupt ER localization to spines.

Journal of Cell Science • Supplementary information

Figure S4

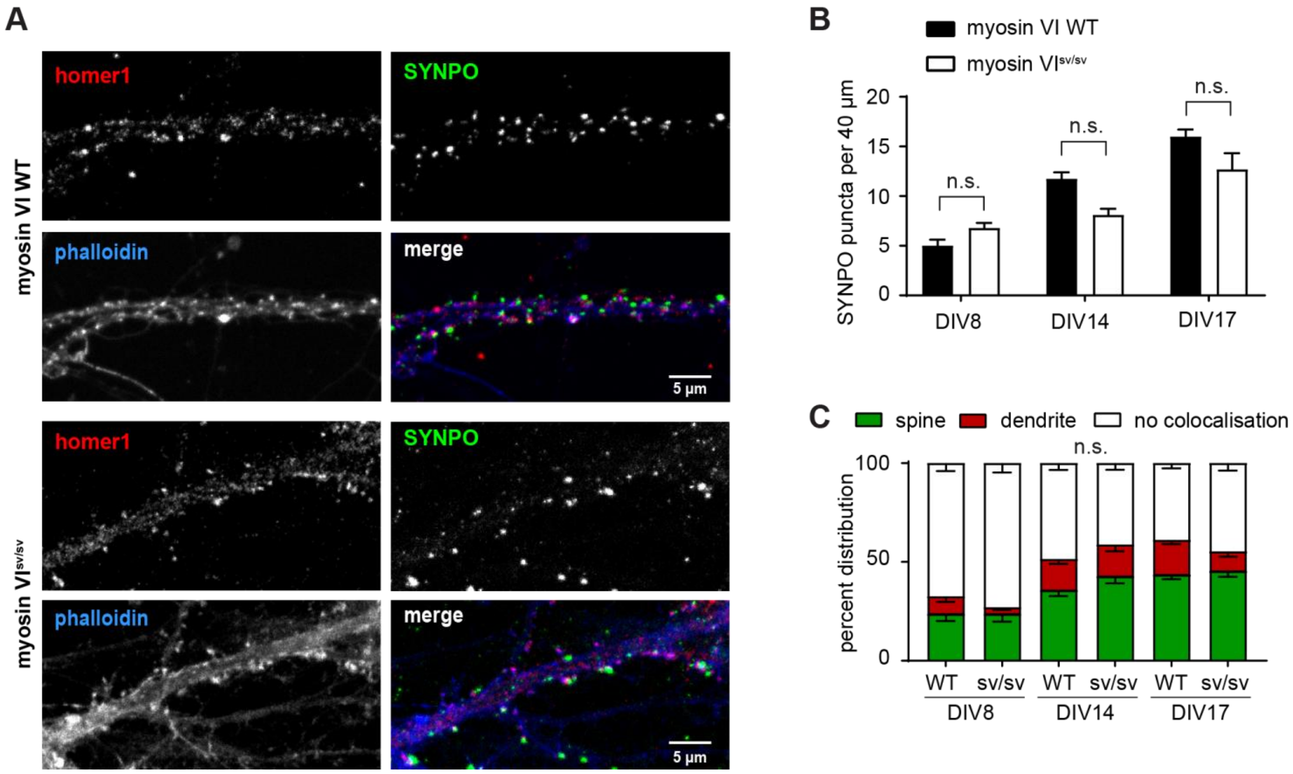

Figure S4. Myosin VI activity is not required for correct localization of synaptopodin

(A) Representative confocal image of primary hippocampal neurons on DIV17 from wild type (WT) and myosin VI-deficient (*Myo6$^{sv/sv}$*) mice stained with anti-synaptopodin, anti-homer1 and phalloidin-A647N. Scale bar = 5 µm.

(B) Quantification (mean ± SEM) of the number of total synaptopodin puncta per 40 µm dendritic segments in WT and *Myo6$^{sv/sv}$* neurons. Two-Way ANOVA, wt vs ko p=0.1364 (n.s.).

(C) Quantification (mean ± SEM) of the percentage of synaptopodin puncta colocalizing with homer1 inside dendritic shafts or spines in WT and *Myo6$^{sv/sv}$* cultures. Mixed ANOVA with DIV as between and localization as within group factors shows no significant differences. F(2, 22)=1.3869, p=0.271. (B+C) All data comes from two independent cultures. DIV8 WT: n=7 cells with 30 segments counted. DIV14 WT: n=8 cells with 32 segments counted. DIV17 WT: n=8 cells with 47 segments counted. DIV8 KO: n=6 cells with 23 segments counted. DIV14 KO: n=6 cells with 31 segments counted. DIV17 KO: n=8 cells with 28 segments counted.

Journal of Cell Science • Supplementary information

Figure S5

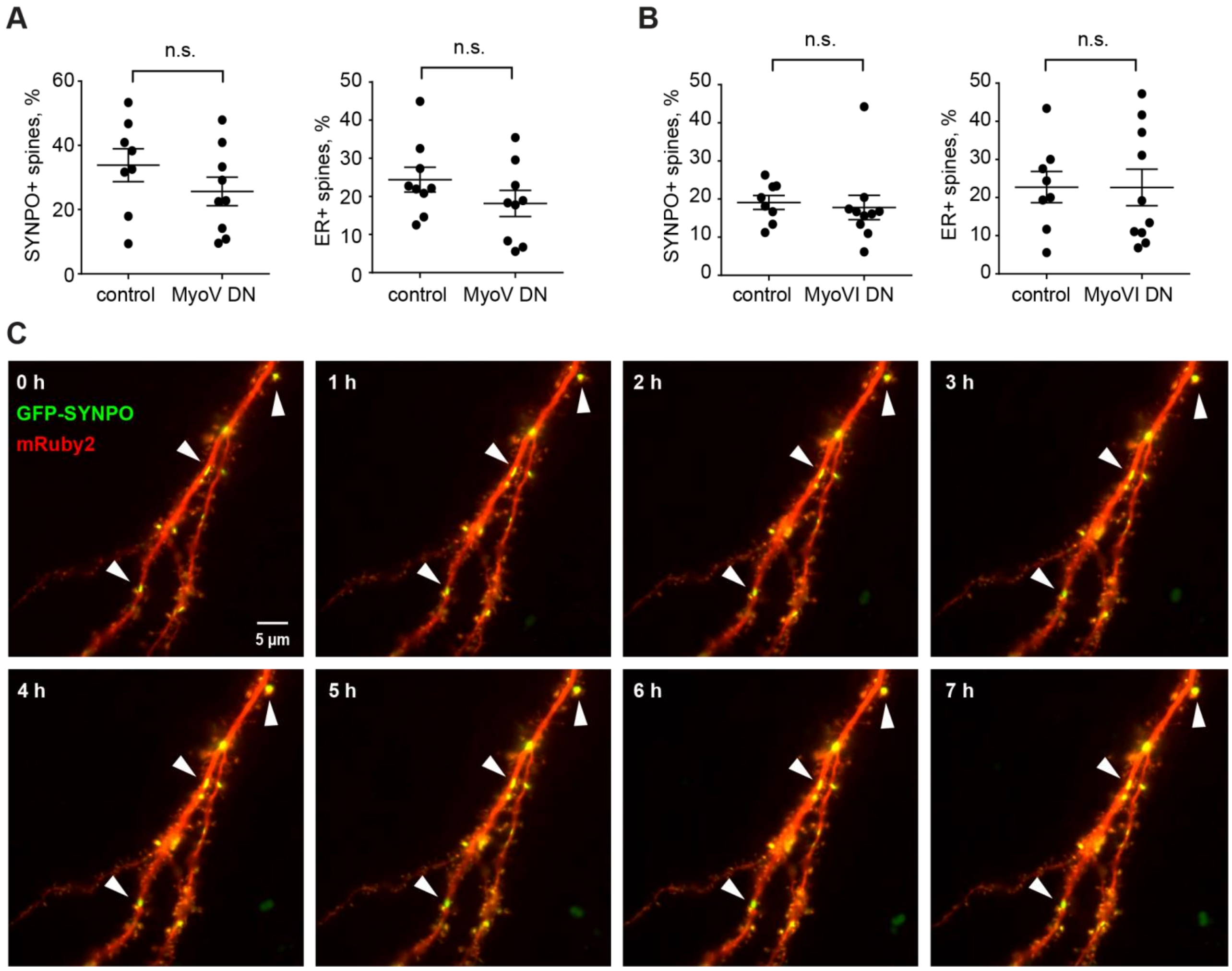

Figure S5. Synaptopodin puncta are stably anchored over long periods of time (> 7 hours) and show no long-distance trafficking events.

(A) Quantification (mean ± SEM) of the percentage of spines that are synaptopodin positive (left), and the number of ER-positive spines (right) for control and MyoV DN cells.

(B) Quantification (mean ± SEM) of the percentage of spines that are synaptopodin positive (left), and the number of ER-positive spines (right) for control and MyoVI DN cells.

(C) Time-lapse imaging of a primary hippocampal neuron (DIV15) transfected with mRuby2 and GFP-synaptopodin followed over 7 h with 5 min intervals. Shown are frames 1 h apart, no changes were observed between these timepoints. Arrowheads indicate examples of GFP-synaptopodin puncta that were stable over the entire imaging period. Scale bar = 5 µm. Also see Movie 1.

Supplementary Movies

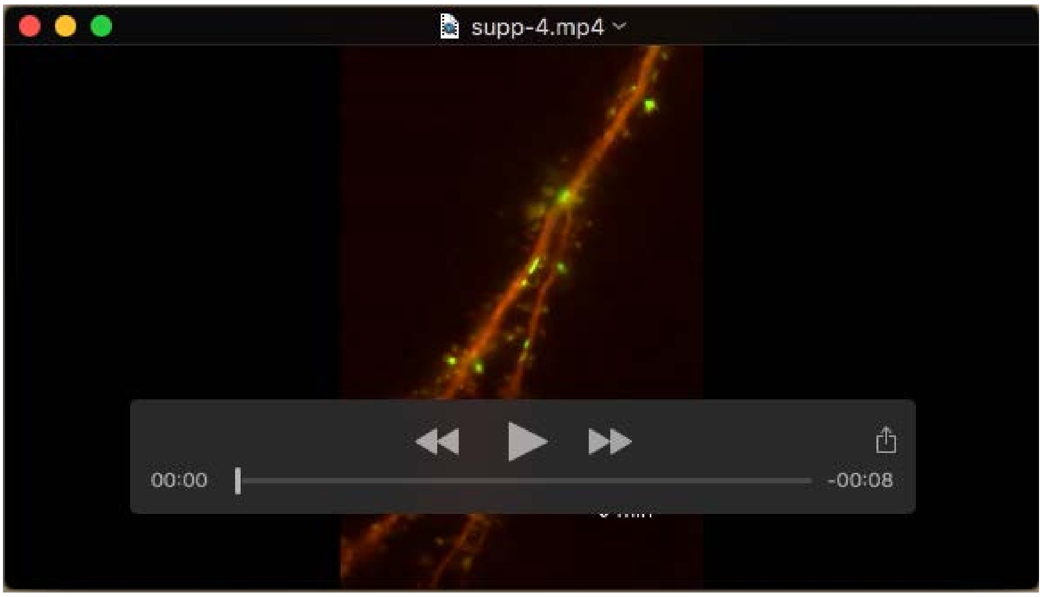

Movie 1. Corresponds to Figure S5. Time-lapse imaging of DIV 17 primary neuron transfected with mRuby2 and GFP-synaptopodin. Movie is acquired with 5 min interval and played at 10 frames per second.

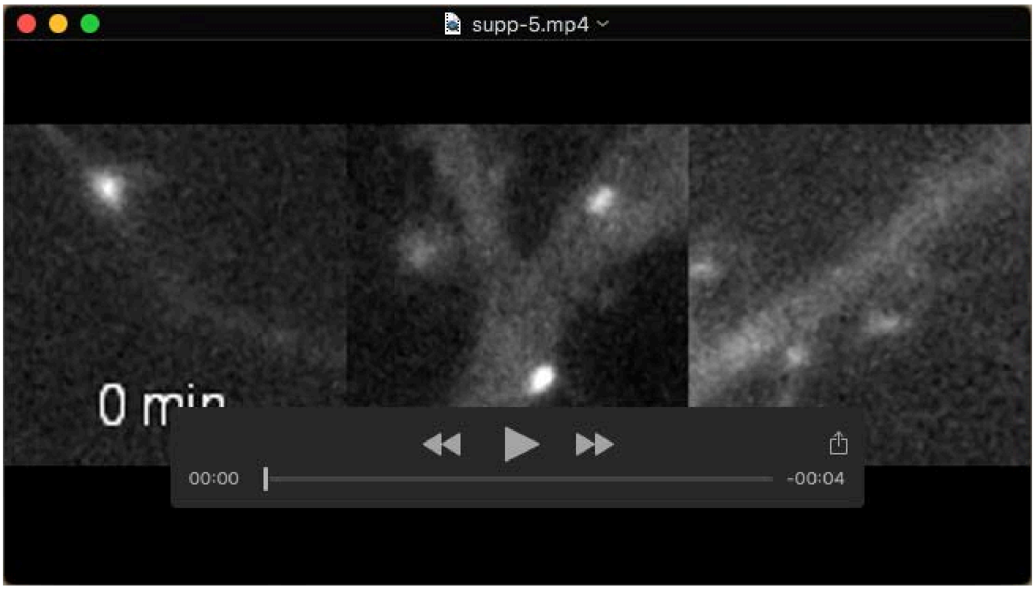

Movie 2. Corresponds to Figure 3B. Time-lapse imaging of DIV17 primary neuron transfected with GFP-synaptopodin. Movie is acquired with 5 min interval and played at 2 frames per second.

Journal of Cell Science • Supplementary information

Supplementary tables

Table S1. Results of mass spectrometry analysis for brain-specific interaction partners of bioGFP-synaptopodin and bioGFP as a control. The table contains hits for proteins from both *Homo sapiens* (coming from HEK cells) and *Rattus norvegicus* (from rat brain lysate). In the following analyses we considered only the proteins from rat origin as those were brain specific. Highlighted in green are unique peptide numbers identified in the bio-GFP-synaptopodin pulldown, and in blue unique peptide numbers from the control bio-GFP pulldown. Highlighted in yellow are protein hits that were used as input for the STRING analysis (Figure 1B, Table S2). Synaptopodin itself is highlighted in red, contaminations (keratins) in light grey.

Click here to Download Table S1

Table S2. Enriched gene ontology terms found in the list of synaptopodin interaction partners from Table S1 (highlighted in yellow) generated with the STRING online analysis tool.

Click here to Download Table S2

Table S3. Expression constructs used in this study.

Backbone	Promoter	Insert	Source
pAAV	synapsin	mRuby2	Addgene #99126 (Chan et al., 2017)
pAAV	synapsin	synaptopodin-GFP	This study
pAAV	synapsin	myosinVI(3177-3789)-DN-GFP	This study
pMH4	synapsin	ER-pDsRed2 (CALR-DsRed-KDEL)	Gift from T.G. Oertner
pCI	synapsin	tdimer2	(Campbell et al., 2002)
pMH4	synapsin	ER-GFP	(Holbro et al., 2009)

Journal of Cell Science • Supplementary information

pCI	synapsin	mCerulean	(Wiegert et al., 2018)
pCI	synapsin	myosinV-DN-mCerulean	This study
pCI	CMV	HA-BirA	(Jaworski et al., 2009)
pEGFP	CMV	synaptopodin-GFP-bio	This study
pL7	L7	empty	(Wagner et al., 2011b)
pL7	L7	mCerulean	(Wagner et al., 2011b)
pL7	L7	mRFP-ER-IRES-GFP	(Wagner et al., 2011b)
pL7	L7	mCER-Myo5a-CC-GTD	This study
pL7	L7	mCER-Myo5a-GTD	This study
pL7	L7	mCER-LZ-Myo5a-GTD	This study

Table S4. Antibodies used in this study.

Primary antibodies:

IF = Immunofluorescence. WB = western blot.

**Antibody**	**Species**	**Source**	**Product #**	**Dilution**
Synaptopodin	Rabbit	Synaptic Systems	163002	IF 1:600 (Fig. 1-5)
Synaptopodin	Mouse	Synaptic Systems	n.a.	IF 1:500 (Fig. S1)
Synaptopodin	Rabbit	Sigma	S9442	IF: 1:1000 (Fig. S1)
MAP2	Mouse	Sigma	M4403	IF 1:500 (Fig. 2;3; S2;S4)
MAP2	Rabbit	Abcam	Ab32454	IF 1:500 (Fig. S2)
homer1	Mouse	Synaptic Systems	160011	IF 1:500 (Fig. 2;4;S4)
myosin Va	Rabbit	Sigma	m4812	WB/IF: 1:500 (Fig. 1)
myosin Vb	Rabbit	Santa Cruz	sc-98020	WB: 1:500 (Fig. 1)
myosin Id	Rabbit	Santa Cruz	sc-66982	WB 1:200 (Fig. 1)
myosin VI	Rabbit	Santa Cruz	sc-50461	WB/IF: 1:200 (Fig. 1)
β-actin	Mouse	Sigma	A5441	WB: 1:500 (Fig. 1;2)
Shank3	Guinea pig	Synaptic Systems	162304	IF: 1:500 (Fig. 1)
GFP	Mouse	Covance	MMS-118P	WB: 1:500

Journal of Cell Science • Supplementary information

Secondary antibodies:

Antibody	Conjugation	Source	Product #	Dilution
anti-rabbit	Alexa Fluor 488	Life Technologies	A11034	IF 1:500
anti-rabbit	Alexa Fluor 586	Life Technologies	A11036	IF 1:500
Anti-rabbit	Alexa Fluor 647	Life Technologies	A21245	IF: 1:500
anti-mouse	Alexa Fluor 488	Life Technologies	A11029	IF 1:500
anti-mouse	Alexa Fluor 586	Life Technologies	A11004	IF 1:500
Anti-guinea pig	Alexa Fluor 488	Invitrogen	A-11073	IF: 1:500
Anti-mouse	AbberiorStar 580	Sigma	**52403**	IF: 1:500 (STED)
Anti-rabbit	Atto-647N	Sigma	**40839**	IF: 1:500 (STED)
anti-rabbit	HRP	Dianova	111-035-144	WB 1:20000
Anti-RFP-X4	AbberiorStar 580	NanoTag	N0404-Ab580-S	ICC: 1:250

Table S5. Pharmacological components.

Reagent	Source	Product number	Final concentration
MyoVin	Merck	475984	30 µM
TIP	Sigma-Aldrich	19566	4 µM
phalloidin–Atto647N	Sigma-Aldrich	65906	IF: 1:40
Streptavidin-HRP	Pierce	21130	WB: 1:10000
DMSO	Carl Roth	4720.4	n.a.
Lysotracker Green	Thermo Fisher	L7526	1:20.000
